# Supplementary material for: Calcium isotope evidence for early Archaean carbonates and subduction of oceanic crust
Source: Nat Commun. 2021 May 5;12:2534. doi: 10.1038/s41467-021-22748-2 (PMC8099908; doi:10.1038/s41467-021-22748-2)
Supplement: Supplementary file 1 — Supplementary Information [file 41467_2021_22748_MOESM1_ESM.pdf]

Supplementary Information

Calcium isotope evidence for early Archaean carbonates and subduction of oceanic crust

Michael A. Antonelli*, Jillian Kendrick, Chris Yakymchuk, Martin Guitreau, Tushar Mittal, Frédéric Moynier

*Correspondence to: Mantonelli@berkeley.edu

This PDF file includes:

I. Supplementary Notes

1 - Sample descriptions

1a – Modern adakite samples

1b – Archean samples

2 - Phase equilibrium modeling

3 - Ca isotope reduced partition function ratios

4 - Tectonic regimes and geothermal gradients in modern oceanic plateaus and adakites

4a - Oceanic plateaus

4b - Modern adakites

5 – Plagioclase fractionation and accumulation

6 - Mixing models

6a - Modern carbonates

6b - Eoarchean seawater/carbonates

7 - Stable calcium *vs.* silicon isotope constraints

II. Supplementary References

III. Supplementary Figures

IV. Supplementary Tables

I. Supplementary Notes

1. Sample Descriptions

1a. Modern adakite samples – Four adakitic samples were selected from an available sample set[1] and collected from three volcanoes in the Austral volcanic zone (AVZ) of the southern Andes. These include two samples from the Burney volcano (Burney 110290-2 and Burney 131285-4), one sample from the Reclus volcano (Reclus 280290-1), and one sample from the Viedma volcano (Viedma 201289-2), which are the same adakitic samples analyzed for Si isotopes[2]. These samples typically have dacitic compositions (SiO_2 ~65 wt%), enrichments in ^{230}Th, and high Sr/Y and La/Yb ratios, implying that they were produced through partial melting of hydrated oceanic crust in equilibrium with an eclogitic residue[1,3]. Although oceanic crust in modern subduction zones generally experiences dehydration (as opposed to melting) due to low geothermal gradients, the subducted crust in the AVZ is young (18-24 Ma) and hot, which allows for partial melting of the subducted slab and production of adakitic lavas[3]. The samples have δ^{18}O values ranging from +6.3 to +7.9‰[3], which is slightly higher than felsic melts derived from a basaltic source in equilibrium with an eclogitic residual mineral assemblage [δ^{18}O ≈ +6.3‰[4]], and could potentially indicate a small contribution from altered (low-temperature) oceanic crust and/or sediments, δ^{18}O increases of up to ~1.5‰, however, are possible during differentiation of hydrous magmas[5].

1b. Archean samples – The TTG samples (n = 18) and tholeiitic granitoid (n = 1) in this study are from a much larger sample set (>140 samples) and were selected based on minimal petrographic evidence for alteration and indistinguishable values for initial hafnium isotopic compositions in zircons and whole-rocks[6]. The samples have available U-Pb geochronology, Sm-Nd, and Lu-Hf isotope data[6], and are the same as those analyzed for Si isotopes[2]. The 19 selected samples have ages ranging from ~4.0 to ~2.8 Ga and come from five different cratons. Four samples (3-85, 74-2, H392, and H395) come from the Baltic shield (collected in Russia, Norway, and Finland), three samples (AG-09-008, AG-09-009, and AG-09-016) come from the Slave craton (Acasta gneiss complex, AGC, Canada), two samples (INO5003 and INO5012) come from the Superior Province (Nuvvuagittuq supracrustal belt, NSB, Canada), four samples (KV2.1/08, NLS5.1-08, ST-J6, and STEY1.5-08) come from the Kaapvaal craton (Barberton greenstone belt, South Africa), one sample (GRO4057) comes from the island west of Innersuartuut (Western Greenland), and five samples (28-03, 40-3, 58-03, 60-04 and 65-10) come from the Tungus-Anabar shield of the Sharyzhalgay uplift (central Siberia). All of these samples are typical TTGs,

except AG-09-008, which is a tonalitic gneiss from the AGC [collected from a unit equivalent to the Idiwhaa unit[7,8]], and most likely represents the product of extensive fractional crystallization of a tholeiitic melt[6]. The TTG samples have SiO_2 of 65-74 wt% with CaO ranging from 1.6 to 5.3 wt% and Sr/Y ratios ranging from ~10 to ~300 (Supplementary Data 1). Detailed petrographic descriptions of the samples are available in Supplementary Data 2.

2. Phase equilibrium modeling

Phase equilibrium modeling for depleted and enriched Archean tholeiite protoliths (DAT and EAT, respectively[9]) is performed in the Na_2O–CaO–K_2O–FeO–MgO–Al_2O_3–SiO_2–H_2O–TiO_2–Fe_2O_3 (NCKFMASHTO) chemical system using the Thermocalc software package, including Thermocalc version 3.45[10] and the thermodynamic dataset[11] (updated version ds62) with results for relevant parameters tabulated in Supplementary Data 4. The activity–composition (*a*–*x*) models used are those of the metabasite set (from May, 2016), including: tonalitic melt, amphibole, clinopyroxene[12], orthopyroxene, garnet, biotite, muscovite, ilmenite[13], K-feldspar, plagioclase[14], and epidote[11]. All these phases have chemical compositions that depend on pressure, temperature, and bulk chemical composition of the system. Rutile, quartz, titanite, and aqueous fluid (H_2O) are modeled as pure phases.

Phase proportions and compositions are calculated along three *P*–*T* gradients, 500°C/GPa, 750°C/GPa, and 1000 °C/GPa, beginning at the modeled solidus for each composition and ending at 1050°C. For each gradient, the H_2O content of the bulk composition is modified to minimally saturate the system in H_2O at the solidus[9]. For the closed system scenario, the bulk composition is fixed along each gradient. The garnet fractionation scenario simulates sequestration of garnet cores from the bulk system, by removing 4.5 mol% of garnet from the bulk composition (major and trace elements) each time 5 mol% is reached, resulting in evolving bulk system compositions along each gradient[9].

The concentrations of trace elements in the modeled melt (Figs. 2b, Supplementary Figs 3, 4, Supplementary Data 4) were calculated using the batch melting equation of Ref. 15, with phase proportions from phase equilibrium modeling and mineral-melt partition coefficients appropriate for partial melting of metabasite[16]. Greater amounts of detail regarding the phase equilibrium modeling in this study can be found in Ref. 9.

3. Reduced partition function ratios for minerals and melt

Equilibrium stable isotope partitioning between phases is determined by their reduced partition function ratios (RPFR, 'β'), which quantify the affinity for heavier Ca isotopes within a single phase[17,18]. At equilibrium, heavy Ca isotopes are favored in phases with stronger bonding environments, such that minerals with shorter and more highly coordinated Ca-O bonds (at a given bond-length) are preferentially stabilized through the incorporation of heavy Ca isotopes. For a majority of mineral phases, we use density-functional theory (DFT) predictions based on generalized gradient approximations (GGA, Supplementary Table 2)[19]. These calculations predict the vibrational frequency shifts and consequent Gibbs free energy decreases in mineral structures upon substitution of a heavy Ca isotope[19]. The DFT predictions appear robust and have been corroborated by (i) triple-Ca isotope measurements (Δ^{48}Ca') in natural plutonic samples[19] and (ii) *ab-initio* estimates using different computational approaches [local-density approximations, LDA[20–23]]. Although large kinetic Ca isotope fractionations have been found in high grade metamorphic rocks[19], metasomatized peridotite[24,25], and rapidly crystallized volcanic samples[26], our current understanding of Ca isotopic equilibration in high-temperature settings suggests that inter-mineral isotopic equilibrium is often reached in slow-cooled plutonic systems[19,26,27], such as those that are targeted in this study.

Importantly, garnet is predicted to be the Ca-bearing mineral phase with the highest equilibrium δ^{44}Ca values. Many trace-element ratios lead to ambiguity between residual garnet (higher-pressures) and residual hornblende (lower-pressures) in the source rocks, and to large debates regarding the depths of TTG petrogenesis[28], Ca isotopes thus provide a unique advantage over trace-element proxies because the isotopic equilibrium values for the two minerals are significantly different[19,20,27,29].

The RPFRs used for the various mineral phases and melt in our models (at 1000 K) can be found in Supplementary Table 2. For garnet, we use predictions for almandine with Ca/(Ca+Fe) = 1/12[19], which are intermediate to those for pyrope (isotopically heavier) and grossular (isotopically lighter), and typically represents the dominant garnet solid-solution species in our model results. Although dilution effects (*e.g.* the amount of Ca and other cation species within a solid-solution mineral) have been observed to change the bonding environments for Ca in pyroxenes, these effects have not been predicted for garnet[19] and are unlikely to play a significant role in our models. Recent modeling work suggests that Ca-O bonds in garnet decrease in length with increasing

pressures (up to 7 GPa), leading to slightly increasing garnet RPFRs[30]. These estimates, however, were not incorporated into our models because the effects are poorly constrained in multi-mineral systems. Preferentially decreasing Ca-O bond lengths in garnet (relative to other mineral phases), however, would serve to increase the predicted Ca isotope fractionations in our models, but these variations are likely to be negligible at the *PT* conditions relevant to TTGs. Plagioclase solid-solution compositions in our model results are typically more albitic, so we use the *ab-initio* predictions for labradorite (An_{50})[19]. For clinopyroxene, we use estimates for diopside[19], and note that the dilution effects in pyroxene solid-solution species[19,22,23] are likely to cancel each other out in our models (*e.g.* higher Fe contents would decrease the RPFR, whereas lower Ca contents would increase the RPFR). Orthopyroxene in our models is based on *ab-initio* models for enstatite with Ca/(Ca+Mg) of 1/64, which are indistinguishable from those for Ca/(Ca+Mg) of 1/32[19,22] and encompass the Ca concentrations of orthopyroxene from our model results.

Using the roughly linear relationships between Ca-O bond lengths and RPFRs at different coordination numbers[27], the bond length and coordination estimates for hornblende [8-fold coordination, 2.474 Å[31,32]] suggest that RPFRs for hornblende are essentially the same as those for diopside. This prediction has also been empirically corroborated by measurements in natural igneous and metamorphic samples[19,26,29]. Similarly, bond-length and coordination constraints for epidote [8-fold coordination, 2.58 Å[33]] suggest that RPFRs are similar to those for pure anorthite [27,34]. Ca isotopes in titanite have only been measured in a single granodiorite sample[35], but bond-length and coordination estimates [7-fold coordination, 2.45 Å[36,37]] suggest that RPFRs for titanite are similar to those predicted for lime[19,27].

Although there are no first-principles predictions for silicate melt, $\delta^{44}Ca$ values for melt/glass are always found to be intermediate to those for clinopyroxene and plagioclase[27], which is true in both mafic[38] and evolved silicic[26] igneous systems. We therefore set the RPFR of silicate melt half-way between the values for diopside and labradorite (An_{50})[19]. Due to the uncertain nature of bond lengths in muscovite and biotite, along with their negligible Ca concentrations, we assume that micas have no effect on mineral-melt fractionation by setting their RPFR values to those of silicate melt.

4. Tectonic regimes and geothermal gradients in modern oceanic plateaus and adakites

The geothermal gradient constraints predicted through our Ca isotope models (500-750ºC/GPa) are typically lower than those predicted at the base of thick oceanic plateaus [>700ºC/GPa[39]] and, along with geochemical evidence for sediment incorporation, suggest that TTGs were produced through subduction of hydrous basaltic crust and sediments along geothermal gradients similar to those predicted for adakites[40–42] and modern (hot) subduction zones[43]. However, it is important to understand the nature and variability of geothermal gradients in the various settings that have been proposed for TTG petrogenesis. In the following section, we provide a short review of modern geophysical observations for oceanic plateaus and adakitic magmatism and discuss these in the context of our isotopic and geochemical constraints for TTG petrogenesis.

4a. Modern observations of oceanic plateaus – Large igneous provinces (LIPs), which include both continental flood basalts as well as oceanic plateaus, can serve as modern analogs for the eruption and emplacement of large magma volumes associated with mantle upwellings on the early Earth. Among the LIPs, the Cretaceous Ontong Java Plateau (OJP) is the most voluminous (> $5x10^6$ km^3) known sequence of basaltic magmatism[44,45]. Typically, oceanic crust with overlying LIPs is 2-5 times thicker than average oceanic crust [~ 7 km[46]], with the OJP having the deepest known Moho depth [30-40 km[47,48]]. This thickness is comparable to that of typical continental crust and of Moho depths under continental flood basalts[49] and could potentially provide the appropriate pressures (~1 GPa) for TTG formation.

The best constraints on extrusive basalt thickness in these settings (~ 4 km) come from an obducted OJP section in the Solomon Islands[50]. Seismic and gravity observations suggest that the rest (20-30 km) of LIP crust is composed of subvolcanic/gabbroic intrusions and magmatic plumbing systems that feed surface eruptions[46,49,51,52]. Geochemical evidence for polybaric crystal fractionation (from ~1 to 0.1 GPa) in erupted basalts[53–55] also suggest a significant role for intrusive plutonism in the evolution of oceanic LIP crust.

A common feature of all oceanic LIPs is that there is a dense [high P-wave velocity (> 7.1 km/s)] lower crustal layer with a typical thickness of at least 5-10 km[46,49,52]. This layer is generally interpreted to represent olivine and pyroxene cumulates formed during fractionation of primary mantle melts at Moho depths[46,56]. These geophysical observations in both oceanic and continental LIPs illustrate that mantle plume-head melting does not typically lead to complete delamination of ultramafic lower crust (even in the largest modern LIPs), which, given that delamination of

lower crustal cumulates is often invoked in order to produce TTGs in oceanic plateau settings[16], casts doubt on the viability of this setting for Archean TTG petrogenesis.

The velocity structure of OJP lithosphere (maximum depths of ~280 km) shows multiple sharp contrasts[57,58] that have been interpreted as structures inherited from oceanic lithosphere atop of which OJP basalts were emplaced. The similar depth of these lithospheric velocity features, compared to similarly-aged normal oceanic lithosphere, thus indicates that the formation of OJP was probably not associated with large scale lithospheric/lower-crustal delamination or significant vertical lithospheric flow in the mantle[57,58].

Additionally, recent numerical models that incorporate effects from density-stratification in oceanic plateau crust[59] show that the upper oceanic crust (hydrated extrusive basalts that are required to explain TTG geochemistry) cannot be transported to Moho depths due to its lower densities and higher viscosities (lower temperatures) compared to those of lower crustal cumulate layers [*e.g.* gravity inversions[60]]. Furthermore, crustal thermal models show that, if oceanic plateaus have a significant intrusive component (*e.g.* intrusive-to-extrusive ratio of ~1), the lower crust (30-40 km depth) is typically hot (800-1200ºC) due to the emplacement of slow-cooling lower crustal intrusions[59], and thus suggest that geothermal gradients in these settings are typically higher than ~800ºC/GPa.

Geothermal gradient estimates from Iceland, where the base of the seismogenic crust (~600-900ºC) is between 10-20 km[61–63], also give apparent geothermal gradients (> ~900ºC/GPa) that are too high for our TTG constraints. Petrological estimates of the *PT* conditions for erupted Icelandic magmas [~1230°C at ~ 0.8 GPa[64]] corroborate this estimate. Even for the thickest Icelandic crust (~ 44 km), seismic observations and petrological modeling indicate that the bottom 15-20 km crust has temperatures greater than 1000ºC[65]. Thus, the geothermal gradient of the most voluminous known mantle-plume/ridge interaction is much higher than suggested by our results for TTGs. Models arguing for an Iceland-type setting for generation of the majority of TTGs[7], therefore, are difficult to reconcile with our observations. We acknowledge, however, that if TTGs formed during the initial stages of LIP construction (*e.g.* during the initial melting phase, when temperatures first exceed ~700ºC at > 1 GPa), this could potentially be consistent with our Ca isotope estimates. Yet, this scenario is unlikely, given that typical crustal temperatures are much hotter during the majority of oceanic plateau formation and eruption.

Although *PT* estimates for peridotite/pyroxenite xenoliths [770-1340ºC at 1.6-3.6 GPa[66,67]] observed in the obducted section of OJP crust[67,68] are similar to those predicted by our Ca isotope constraints, no TTG-like granitoids have been observed in the OJP. This further suggests that hydrated basaltic crust cannot be transported to appropriate depths for TTG formation in modern oceanic plateaus. More efficient vertical transport, however, may have been promoted by higher mantle potential temperatures in the Archean[69,70], but it would still be difficult to transport high-viscosity upper oceanic crustal basalts to sufficient depths for TTG formation[59]. Recent models, on the other hand, suggest that thick Archean oceanic crust was less, not more, buoyant than modern crust[71], facilitating the early operation of subduction-driven plate tectonics. We conclude that some form of subduction - either analogous to modern plate tectonics, or of a more episodic style[72–74] – coupled with vertical deformation due to crustal stresses[75] provides the most parsimonious explanation for our data.

4b. Subduction and modern adakite petrogenesis – Adakites represent one of the closest modern analogs of Archean TTGs, so understanding their geodynamic settings on modern Earth can provide important constraints on the settings that generated ancient TTGs [see[40] and reference therein]. Adakites, especially those with compositions closest to TTGs, are typically associated with subduction of either (i) a hot/young oceanic plate [*e.g.* Austral volcanic zone[3]], (ii) a buoyant oceanic plateau [*e.g.* Jamaica-type adakites[40,76]], or (iii) a mid-ocean ridge [leading to the formation of a slab window[41,42,77,78]]. In addition to melting triggered by aesthenospheric upwelling through slab-windows[79,80], toroidal mantle flow around the edges of a subducting flat slab [*e.g.* Yakutat oceanic slab in Alaska and Wrangell volcanics[81,82]] could also lead to production of adakitic magmas. In almost all of these cases, however, adakitic melts are formed through partial melting of subducted hydrated basaltic crust (± sediments). Other than through potential differences in protolith compositions and geodynamic settings, various degrees of melt-percolation through thin mantle wedges and/or fractional crystallization before eruption can also generate some variability[40,41,77,83–85].

There are some adakites, however, that are not-associated with active subduction settings, which can be generated through mantle upwelling due to crustal extension or plume activity [*e.g.* Borneo[86], Kamchatka[87]]. Nevertheless, these settings also require melting of hydrated basaltic crust. In almost all models for generation of adakites, therefore, subduction processes are required either directly (*e.g.* slab melting) or at some time in the past, such that hydrated basalts and

sediments were transported to sufficient depths for later melting at amphibolite/eclogite facies conditions. Although a large majority of models for adakite petrogenesis require hydrated basaltic source-rocks, we note that some models suggest that melting of subducting *lower* crust [metagabbros, which are analogous to the dense cumulate lower crustal layer in LIPs] can also lead to tonalite-trondhjemite magmatism, either due to ridge subduction or subduction-erosion/crustal delamination[41,88–91]. Our Ca isotope data, along with Si isotope data in previous studies[2,92], however, support models where low-temperature oceanic sediments are included into TTG source rocks, and thus indicate that hydrated basaltic oceanic crust is likely to have been the dominant protolith for TTGs in the Archean.

5. Plagioclase fractionation and accumulation

As mentioned in the main text, plagioclase is the only Ca-bearing silicate predicted to be isotopically lighter than melt at equilibrium[26,27,38]. Plagioclase fractionation, therefore, is the only equilibrium magmatic process able to drive melt $\delta^{44}Ca$ higher than BSE (> 0‰). The three TTG samples with $\delta^{44}Ca$ > 0‰ (up to +0.1‰) have small negative Eu-anomalies [$Eu/Eu^* = Eu/(Sm \times Gd)^{0.5} < 1$] down to ~0.7 also indicative of plagioclase fractionation. Although we have not included fractional crystallization processes into our phase equilibrium models, we can place rough constraints on the amount of plagioclase crystallization that is needed to explain the sample compositions using a Rayleigh fractionation model (Supplementary Figure 5). Assuming that $\alpha_{plag\text{-}melt}$ is 0.99985 (T = 800ºC), $K_d(Ca) = 4$ and $K_d(Eu) = 2$ [distribution coefficients for Ca and Eu, respectively, for plagioclase in equilibrium with dacitic melt[93–95]], and that melt starts with Eu/Eu* = 1 and $\delta^{44}Ca$ = -0.1‰ (as predicted for the high dT/dP case in our phase-equilibrium models), we find that a positive shift of +0.2‰ for $\delta^{44}Ca$ and negative shift in Eu/Eu* (down to ~0.7) can be explained by 30-40% fractional crystallization of plagioclase (Supplementary Figure 5b). This represents a Ca fraction of ~0.25 in the remaining liquid (Supplementary Figure 5a). Although the signals are most obvious in the three samples with $\delta^{44}Ca$ > 0, six other samples also have Eu/Eu* lower than predicted by our phase-equilibrium models (down to ~0.5, Supplementary Figure 3), suggesting that they also underwent some degree of plagioclase fractionation during crystallization and that their $\delta^{44}Ca$ values may have increased by up to ~0.3‰ (up to ~50% fractional crystallization, with a residual Ca fraction in the liquid down to ~0.15). Although these calculations are rough approximations at best, the results suggest that our geothermal gradient estimates are

mainly upper limits, as the original melt δ^{44}Ca values, prior to plagioclase crystallization, were likely to have been more negative. Although we do not attribute the formation of Archaean TTGs solely to fractional crystallization[96,97], our data suggest that fractional crystallization of plagioclase may have played a role in their geochemical evolution, after their initial formation through partial melting of hydrated oceanic crust.

Two of our TTG samples, however, have positive Eu-anomalies (~2.4 and ~7.8) that suggest plagioclase accumulation. Using the same Ca and Eu distribution coefficients, $\alpha_{plag\text{-}melt}$, and starting conditions as for the plagioclase fractionation calculations, we place rough constraints on the amount of plagioclase accumulation necessary to explain Eu/Eu* of the crystalized (pooled) solids. Although these estimates are complicated by the contributions from other minerals in the cumulates (and changing temperatures during crystallization), plagioclase is the most abundant Ca bearing mineral in TTGs, so the effect of other minerals on the δ^{44}Ca of the pooled product should be relatively negligible. A Eu/Eu* value of ~2.4 in the pooled products represents ~20% fractional crystallization of plagioclase, which would result in a negative δ^{44}Ca shift of -0.10‰. A Eu-anomaly of ~7.8 represents the pooled product for ~2% fractional crystallization of plagioclase, which leads to a negative δ^{44}Ca shift of -0.14‰ in the solids. This suggests that the measured δ^{44}Ca in these two samples may be slightly lower (by ~0.1‰) than in the parental TTG melts from which they crystallized, but the shifts are similar to our measurement uncertainties and do not affect our interpretations.

6. Mixing Models

Given that the two TTG samples from the Nuvvuagittuq Supracrustal Belt (NSB) have δ^{44}Ca too negative to be explained by our magmatic differentiation models, they must have succumb to either (i) kinetic effects during/after emplacement or (ii) mixing with isotopically light Ca in the source materials. Although kinetic effects during crystallization[26] are unable to cause large negative Ca isotope fractionations in bulk magma (see main text), kinetic effects during post-emplacement metamorphism and/or during diffusion of Ca into the melts (*e.g.* during percolation of TTG magmas through a mantle wedge) could be potential mechanisms for decreasing δ^{44}Ca in our samples. Apart from their elevated δ^{18}O values and peraluminosity (A/CNK), however, the TTG samples from the NSB have similar major and trace-element compositions to the other TTGs analyzed in this study. They were also collected from a locality that is petrologically similar to the

other samples (*e.g.* sampled from TTG gneisses with no melanocratic/leucocratic banding, far from surrounding lithologic contacts, Supplementary Note 1, Supplementary Data 2) so the question arises as to why kinetic effects would only have occurred in NSB samples. Indeed, the mobile/immobile trace element ratios (Supplementary Figure 6) and initial ε_{Hf} values that are the same in zircons and in whole rocks[6] suggest that metasomatic/metamorphic processes did not greatly affect the samples on a bulk chemical level. Thus, the low δ^{44}Ca values, along with the high A/CNK and δ^{18}O values [which strongly suggest sediment incorporation[98,99]], are best explained by incorporation of high Ca (carbonate) sediments with low δ^{44}Ca into the NSB magma sources. The Ca isotope compositions of modern and inferred Eoarchaean carbonates are discussed in the following sections.

6a. Modern carbonates – Although the δ^{44}Ca values used for carbonates in different mixing models have varied substantially across the literature[100–107], ranging from -0.35‰ [the average $\delta^{44}Ca_{BSE}$ value for Phanerozoic carbonates[108]] to -0.95‰ (the value of carbonate standard SRM915a), the basis for using lower-than-average δ^{44}Ca has not been explicitly discussed. Precambrian carbonates younger than 3.0 Ga have average δ^{44}Ca values even closer to BSE[109], so, at our current level of understanding, it is not possible to get large δ^{44}Ca effects[110] by mixing in average carbonates.

This suggests that studies where carbonate (or carbonate melt) δ^{44}Ca values lower than -0.35‰ are invoked in order to explain the data[100,101,103,104,107] could be due to (i) unconsidered magmatic differentiation effects, (ii) kinetic isotope effects, or (iii) incorporation of especially light (rapidly-precipitated) carbonates with non-average δ^{44}Ca. Given that the NSB samples have strong evidence for incorporation of sedimentary materials, and that we have carefully considered magmatic differentiation and possible kinetic effects, it follows that the assimilated sediments must have also included Ca-rich materials (carbonates) with δ^{44}Ca values lower than those recorded in the NSB TTGs (< -0.85‰). Although values lower than this represent less than ~5% of modern carbonates[108], coming dominantly from rapid precipitation of aragonite (inducing large kinetic δ^{44}Ca fractionations), such as can occur in shallow shelf settings[111], it could be possible that our NSB samples record such sediments. On the other hand, the limited (and potentially hard-to-subduct) geologic settings that produce such large kinetic effects during carbonate precipitation may cast doubt on this interpretation. As an alternative explanation, a majority of carbonates could

more easily reach values lower than -0.85 ‰ (at average precipitation rates) if δ^{44}Ca of ancient seawater was similar to BSE.

Carbonates within altered oceanic crust (AOC) could also be invoked to explain our data, but these tend to have more positive δ^{44}Ca than sedimentary carbonates[109], with Ca isotope compositions typically between BSE (δ^{44}Ca = 0‰) and modern seawater (δ^{44}Ca = +0.9‰), which could be due to (i) slower precipitation rates and higher temperatures than for sedimentary carbonates and (ii) some of the Ca in AOC carbonates coming from basalts rather than purely from seawater. If the first scenario is correct, then it may be possible that BSE-like seawater with δ^{44}Ca = 0‰ could have yielded AOC carbonates with δ^{44}Ca down to -0.9‰, as required by our NSB samples, on the other hand, any Ca contributions from basalts would dilute this signal (pushing values back towards 0‰). Given the relatively smaller $\Delta^{44}Ca_{SW\text{-}carbonate}$ fractionations associated with AOC carbonates, an AOC origin for carbonates in NSB protoliths would require that (i) essentially all the Ca in the NSB samples came from carbonates and that (ii) these carbonates represented the lowest $\Delta^{44}Ca_{SW\text{-}carbonate}$ end-members observed for AOC[109]. It is more likely, therefore, that smaller amounts of sedimentary carbonates with average δ^{44}Ca values lower than -0.9‰ (e.g., -1.25‰, as suggested in the main text) were incorporated into the NSB protoliths.

Although subduction of carbonate sediments may be more difficult than AOC carbonates, Fe isotope and chemical data from NSB samples also suggest that sedimentary carbonates were present in NSB protoliths[112], while δ^{30}Si data in the same samples analyzed by this study (INO5003 & INO5012) suggest that incorporation of chemical sediments (chert) also occurred[2]. Furthermore, $^{87}Sr/^{86}Sr$ measurements in zircon-hosted apatite from the same locality suggest that the NSB protoliths had high Rb/Sr ratios[113] potentially indicative of clay-rich sediments; and an associated terrane (the Ukaliq Supracrustal Belt, USB, ~3km to the North) has relatively high δ^{18}O and δ^{30}Si values also suggestive of chemical sediments in protoliths[114]. Indeed, the large number of metasedimentary units associated with amphibolites in the NSB region[115–119], which are potential precursors to TTGs in the area[120], are likely to have been assimilated into TTG melts. Together, these observations lend weight to our hypothesis that both terrigenous sediments (shales) and chemical sediments (carbonates) were, at least locally, present in the Eoarchean oceans and were subsequently incorporated into NSB TTGs.

6b. Hadean/early Archean seawater and carbonates – Modern seawater has evolved towards heavier δ^{44}Ca values over the Phanerozoic[121], as a likely result of the increasing amounts of

biological carbonate precipitation (which serves as a sink for isotopically light Ca). Prior to the development of biological carbonate precipitation, however, seawater may have had δ^{44}Ca values closer to bulk-silicate Earth. This effect would also be strongly reinforced by the higher internal heat budget of the early Earth[122,123], which would have caused more pervasive hydrothermal circulation and increased input of hydrothermal Ca into the oceans. Modern end-member hydrothermal vent fluids have δ^{44}Ca = ~0‰[124–126] with basaltic radiogenic Ca (ε_{Ca}) and dominantly basaltic Sr isotope (^{87}Sr/^{86}Sr) signals[27,34,127–129]. Ca from hydrothermal vents, therefore, represents addition of non-fractionated Ca from seafloor basalts. Thus, both higher levels of hydrothermal input and lower levels of biological carbonate precipitation are likely to have buffered Hadean/early Archean seawater at δ^{44}Ca values ≈ 0‰. The prediction for mantle-buffered Archean seawater is not new, and is also supported by paleoseawater chemistry and ^{87}Sr/^{86}Sr data[130,131]. This postulate has important implications, however, because carbonates precipitated from BSE-like (mantle-buffered) seawater would have average δ^{44}Ca of -1.25‰ (assuming the same average $\Delta^{44}Ca_{carb-sw}$ as today), which is low enough to explain our NSB samples.

The question still remains, however, as to whether average modern seawater-carbonate Ca isotope fractionation ($\Delta^{44}Ca_{carb-sw}$, which is fairly large today due to contributions from biological carbonate precipitation) is applicable to early Eoarchean systems. The earliest widely-accepted evidence for biologically-induced carbonate precipitation comes from ~3.5 Ga stromatolites[132,133], though some authors argue that stromatolites may have existed before this, prior to ~3.7 Ga[134–136]. Thus, it is not inconceivable that early Eoarchean $\Delta^{44}Ca_{carb-sw}$ may have included biological carbonates and been similar to modern. On the other hand, abiotic carbonate precipitation [such as aragonite fans precipitated directly onto the seafloor[137]] would have dominated prior to the evolution of organisms capable of promoting carbonate precipitation. Abiotic carbonate precipitation has also been shown to result in $\Delta^{44}Ca_{carb-sw}$ as large as -1.5‰ for rapidly-precipitated calcite[138], and slightly larger for abiotically precipitated aragonite[139]. Regardless of the polymorph and the lack of ancient carbonate units (> 3.8 Ga) preserved today, carbonate precipitation in the Hadean and early Eoarchean is required for maintaining clement surface conditions [as evidenced by liquid water on Earth's surface[140,141]] at a time where high levels of volcanic CO_2 outgassing[142] would have otherwise led to an ever-increasing buildup of atmospheric CO_2 and corresponding run-away greenhouse effect[143].

The multi-proxy evidence for sedimentary material incorporation in NSB samples also has implications for recent work concluding that NSB protoliths contained high Rb and K concentrations suggestive of high-silica Hadean crust[113]. Sediment assimilation in NSB samples could serve as an alternative explanation for their high protolith Rb and K concentrations, but further work is required to better evaluate these hypotheses. Finally, we note that many of the parameters used for the shale and carbonate end-members are close to those observed in low-T altered clays and carbonates from oceanic hyaloclastites[144]. Our mixing models would therefore lead to similar results (*e.g.* ~30-50% assimilation of low-T surface materials) if typical hyaloclastite carbonate/silicate compositions were used as the sedimentary end-members.

Although 30-50 wt% sediment incorporation can be considered high, up to 50% sedimentary materials have been previously suggested to explain Hf and O isotope signatures in granitic magmas[145], and earlier iterations of our phase-equilibrium models suggest that lesser amounts of sediment incorporation (*e.g.* ~20 wt%) cannot produce melts with A/CNK high enough to explain the Nuvvuagittuq samples (A/CNK > 1.3). While physical mechanisms related to sediment incorporation in magmas are beyond the scope of this work, incorporation of previously melted sedimentary materials has been suggested to facilitate the process[146]. Another option could be that low-T altered oceanic crust (*e.g.* hyaloclastites, containing both clays and carbonates, as opposed to marly sediments) may be more resistant to the 'skimming-off' of sediments from the top of the slab during subduction. Incorporation of sedimentary materials during emplacement of TTG plutons could also be invoked to explain the high A/CNK of our NSB samples; yet, assimilation of such large quantities of sedimentary material (30-50 wt%) would be unlikely given that the thermal requirements of assimilation make it a self-limiting process[147], especially in relatively low-temperature granitoid magmas.

7. Stable calcium *vs.* silicon isotope constraints

The stable Ca and Si isotope compositions for AVZ adakites and TTGs are compared in Supplementary Figure 7. In terms of Si isotope fractionation, melts produced at greater depths (with higher amounts of residual garnet) are expected to have higher δ^{30}Si (the opposite of δ^{44}Ca) because garnet has the second-lowest equilibrium δ^{30}Si composition of all major silicate minerals[148–151]. Plagioclase fractionation, which is necessary in order to explain the small group of

TTG samples with $\delta^{44}Ca$ greater than BSE, likely leads to small negative $\delta^{30}Si$ effects, as plagioclase is predicted to have slightly higher $\delta^{30}Si$ than most silicate melts at equilibrium[149].

Magmatic differentiation effects for Si isotopes, however, were considered in[2] and were not found to produce large enough $\delta^{30}Si$ values to explain TTGs. These authors, along with[92], thus concluded that TTG source-rocks must have included sedimentary chert with positive $\delta^{30}Si$. Stable Ca isotopes in TTGs, on the other hand, dominantly record magmatic differentiation processes and, other than in the two NSB samples, do not require addition of carbonate sediments to explain their $\delta^{44}Ca$ values. This is perhaps not surprising due to the different geographic settings for silica (colder/deeper water) and carbonate precipitation (warmer/shallower water) on the modern Earth[152], but does raise the question as to how the Nuvvuagittuq samples record both incorporation of isotopically heavy $\delta^{30}Si$ chert and of isotopically light $\delta^{44}Ca$ carbonates. Clasts and layers of chert are often found in limestone beds, however, and both can be incorporated into mudstone matrix in accretionary prisms[153], which have arguably been identified as early as ~3.7 Ga in Isua supracrustal sequences[154,155]. Alternatively, cherts and carbonates can be mixed together with other deep-sea sediments and basalts in both accretionary and collisional orogens[156,157]. Unlike for Ca isotopes, heavy Si isotope signatures indicative of siliceous sediment incorporation are ubiquitous in TTGs[2,92], which might suggest that the geographic locations amenable to chert precipitation were more abundant than those amenable to carbonate precipitation (or were more likely to be subducted) on the early Earth, or alternatively, that most Eoarchean carbonates had $\delta^{44}Ca$ values similar to average TTGs. In combination, however, the geothermal gradient constraints derived from $\delta^{44}Ca$ variations and the Si isotopic data suggestive of chert incorporation, together imply that sediments were subducted along geotherms (~500-750ºC/GPa) similar to those found for modern hot subduction zones[43], and suggest that subduction-driven plate tectonics was operative by the Eoarchean.

II. Supplementary References

1. Sigmarsson, O., Martin, H. & Knowles, J. Melting of a subducting oceanic crust from U–Th disequilibria in austral Andean lavas. *Nature* **394**, 566–569 (1998).
2. Deng, Z. *et al.* An oceanic subduction origin for Archaean granitoids revealed by silicon isotopes. *Nat. Geosci.* **12**, 774–778 (2019).
3. Stern, C. R. & Kilian, R. Role of the subducted slab, mantle wedge and continental crust in the generation of adakites from the Andean Austral Volcanic Zone. *Contrib. to Mineral. Petrol.* **123**, 263–281 (1996).
4. Bindeman, I. N. *et al.* Oxygen isotope evidence for slab melting in modern and ancient subduction zones. *Earth Planet. Sci. Lett.* **235**, 480–496 (2005).
5. Bucholz, C. E., Jagoutz, O., VanTongeren, J. A., Setera, J. & Wang, Z. Oxygen isotope trajectories of crystallizing melts: Insights from modeling and the plutonic record. *Geochim. Cosmochim. Acta* **207**, 154–184 (2017).
6. Guitreau, M., Blichert-Toft, J., Martin, H., Mojzsis, S. J. & Albarède, F. Hafnium isotope evidence from Archean granitic rocks for deep-mantle origin of continental crust. *Earth Planet. Sci. Lett.* **337**–**338**, 211–223 (2012).
7. Reimink, J. R., Chacko, T., Stern, R. A. & Heaman, L. M. Earth's earliest evolved crust generated in an Iceland-like setting. *Nat. Geosci.* **7**, 529–533 (2014).
8. Mojzsis, S. J. *et al.* Component geochronology in the polyphase ca. 3920Ma Acasta Gneiss. *Geochim. Cosmochim. Acta* **133**, 68–96 (2014).
9. Kendrick, J. & Yakymchuk, C. Garnet fractionation, progressive melt loss and bulk composition variations in anatectic metabasites: Complications for interpreting the geodynamic significance of TTGs. *Geosci. Front.* **11**, 745–763 (2020).
10. Powell, R. An internally consistent dataset with uncertainties and correlations : 3. Applications to geobarometry, worked examples and a computer program. *J. Metamorph. Geol.* 173–204 (1988).
11. Holland, T. J. B. & Powell, R. An improved and extended internally consistent thermodynamic dataset for phases of petrological interest , involving a new equation of state for solids. *J. Metamorph. Geo* 333–383 (2011) doi:10.1111/j.1525-1314.2010.00923.x.
12. Green, E. C. R. R. *et al.* Activity–composition relations for the calculation of partial

melting equilibria in metabasic rocks. *J. Metamorph. Geol.* **34**, 845–869 (2016).

13. White, R. W., Powell, R., Holland, T. J. B., Johnson, T. E. & Green, E. C. R. New mineral activity – composition relations for thermodynamic calculations in metapelitic systems. *J. Metamorph. Geol.* 261–286 (2014) doi:10.1111/jmg.12071.
14. Holland, T. & Powell, Æ. R. Activity – composition relations for phases in petrological calculations : an asymmetric multicomponent formulation. *Contrib. to Mineral. Petrol.* 492–501 (2003) doi:10.1007/s00410-003-0464-z.
15. Shaw, D. M. Trace element fractionation during anatexis. *Geochim. Cosmochim. Acta* **34**, 237–243 (1970).
16. Bédard, J. H. A catalytic delamination-driven model for coupled genesis of Archaean crust and sub-continental lithospheric mantle. *Geochim. Cosmochim. Acta* **70**, 1188–1214 (2006).
17. Young, E. D., Galy, A. & Nagahara, H. Kinetic and equilibrium mass-dependant isotope fractionation laws in nature and their geochemical and cosmochemical significance. *Geochim. Cosmochim. Acta* **66**, 1095–1104 (2002).
18. Urey, H. C. The thermodynamic properties of isotopic substances. *J. Chem. Soc.* 562 (1947) doi:10.1039/jr9470000562.
19. Antonelli, M. A. *et al.* Kinetic and equilibrium Ca isotope effects in high-T rocks and minerals. *Earth Planet. Sci. Lett.* **517**, 71–82 (2019).
20. Huang, F., Zhou, C., Wang, W., Kang, J. & Wu, Z. First-principles calculations of equilibrium Ca isotope fractionation : Implications for oldhamite formation and evolution of lunar magma ocean. *Earth Planet. Sci. Lett.* **510**, 153–160 (2019).
21. Song, Y., Li, Y., Wang, W. & Wu, Z. First-principles investigation of the concentration effect on equilibrium fractionation of Ca isotopes in forsterite. *Acta Geochim.* (2019) doi:10.1007/s11631-019-00346-w.
22. Wang, W. *et al.* Concentration effect on equilibrium fractionation of Mg-Ca isotopes in carbonate minerals: Insights from first-principles calculations. *Geochim. Cosmochim. Acta* **208**, 185–197 (2017).
23. Feng, C., Qin, T., Huang, S., Wu, Z. & Huang, F. First-principles investigations of equilibrium calcium isotope fractionation between clinopyroxene and Ca-doped orthopyroxene. *Geochim. Cosmochim. Acta* **143**, 132–142 (2014).

24. Zhao, X. *et al.* Coupled extremely light Ca and Fe isotopes in peridotites. *Geochim. Cosmochim. Acta* **208**, 368–380 (2017).
25. Kang, J. T. *et al.* Diffusion-driven Ca-Fe isotope fractionations in the upper mantle: Implications for mantle cooling and melt infiltration. *Geochim. Cosmochim. Acta* **290**, 41–58 (2020).
26. Antonelli, M. A. *et al.* Ca isotopes record rapid crystal growth in volcanic and subvolcanic systems. *Proc. Natl. Acad. Sci. U. S. A.* **116**, 20315–20321 (2019).
27. Antonelli, M. A. & Simon, J. I. Calcium isotopes in high-temperature terrestrial processes. *Chem. Geol.* **548**, (2020).
28. Smithies, R. H. *et al.* No evidence for high-pressure melting of Earth's crust in the Archean. *Nat. Commun.* **10**, (2019).
29. Wang, Y. *et al.* Calcium isotope fractionation during crustal melting and magma differentiation: Granitoid and mineral-pair perspectives. *Geochim. Cosmochim. Acta* **259**, 37–52 (2019).
30. Chen, C. *et al.* Compositional and pressure controls on calcium and magnesium isotope fractionation in magmatic systems. *Geochim. Cosmochim. Acta* (2020) doi:10.1016/j.gca.2020.09.006.
31. Hawthorne, F. C. & Oberti, R. Classification of the amphiboles. *Rev. Mineral. Geochemistry* **67**, 55–88 (2007).
32. Zhou, C., Wang, W., Kang, J. & Wu, Z.;Huang, F. First-principles Calculations of Equilibrium Calcium Isotope Fractionation among Ca-bearing Minerals. in *AGU Fall Meeting* (2016).
33. Franz, G. & Liebscher, A. Physical and Chemical Properties of the Epidote Minerals-An Introduction-. *Rev. Mineral. Geochemistry* **56**, 1–81 (2004).
34. Brown, S. T. *et al.* High-temperature kinetic isotope fractionation of calcium in epidosites from modern and ancient seafloor hydrothermal systems. *Earth Planet. Sci. Lett.* **535**, 116101 (2020).
35. Ryu, J. S., Jacobson, A. D., Holmden, C., Lundstrom, C. & Zhang, Z. The major ion, d44/40Ca, d44/42Ca, and d26/24Mg geochemistry of granite weathering at pH=1 and T=25 C: Power-law processes and the relative reactivity of minerals. *Geochim. Cosmochim. Acta* **75**, 6004–6026 (2011).

36. Hollabaugh, C. L. & Foit, F. F. The crystal structure of an Al-rich titanite from Grisons, Switzerland. *Am. Mineral.* **69**, 725–732 (1984).
37. Kunz, M., Arlt, T. & Stolz, J. In situ powder diffraction study of titanite (CaTiOSiO4) at high pressure and high temperature. *Am. Mineral.* **85**, 1465–1473 (2000).
38. Zhang, H. *et al.* No Measurable Calcium Isotopic Fractionation During Crystallization of Kilauea Iki Lava Lake. *Geochemistry, Geophys. Geosystems* **19**, 3128–3139 (2018).
39. Johnson, T. E., Brown, M., Gardiner, N. J., Kirkland, C. L. & Smithies, R. H. Earth's first stable continents did not form by subduction. *Nature* **543**, 239–242 (2017).
40. Hastie, A. R. *et al.* Can Fractional Crystallization, Mixing and Assimilation Processes be Responsible for Jamaican-type Adakites? Implications for Generating Eoarchaean Continental Crust. *J. Petrol.* **56**, 1251–1284 (2015).
41. Castillo, P. R. Adakite petrogenesis. *Lithos* **134–135**, 304–316 (2012).
42. Hastie, A. R., Fitton, J. G., Bromiley, G. D., Butler, I. B. & Odling, N. W. A. The origin of Earth's first continents and the onset of plate tectonics. *Geology* **44**, 855–858 (2016).
43. Penniston-Dorland, S. C., Kohn, M. J. & Manning, C. E. The global range of subduction zone thermal structures from exhumed blueschists and eclogites: Rocks are hotter than models. *Earth Planet. Sci. Lett.* **428**, 243–254 (2015).
44. Clapham, M. E. & Renne, P. R. Flood Basalts and Mass Extinctions. *Annu. Rev. Earth Planet. Sci.* **47**, 275–303 (2019).
45. Hochmuth, K., Gohl, K. & Uenzelmann-Neben, G. Playing jigsaw with Large Igneous Provinces-A plate tectonic reconstruction of Ontong Java Nui, West Pacific. *Geochemistry, Geophys. Geosystems* **16**, 3789–3807 (2015).
46. Ridley, V. A. & Richards, M. A. Deep crustal structure beneath large igneous provinces and the petrologic evolution of flood basalts. *Geochemistry Geophys. Geosystems* **11**, Q09006 (2010).
47. Gladczenko, T. P., Coffin, M. F. & Eldholm, O. Crustal structure of the Ontong Java Plateau: Modeling of new gravity and existing seismic data. *J. Geophys. Res. Solid Earth* **102**, 22711–22729 (1997).
48. Miura, S. *et al.* Seismological structure and implications of collision between the Ontong Java Plateau and Solomon Island Arc from ocean bottom seismometer–airgun data. *Tectonophysics* **389**, 191–220 (2004).

49. Richards, M., Contreras-Reyes, E., Lithgow-Bertelloni, C., Ghiorso, M. & Stixrude, L. Petrological interpretation of deep crustal intrusive bodies beneath oceanic hotspot provinces. *Geochemistry, Geophys. Geosystems* **14**, 604–619 (2013).
50. Petterson, M. G. The geology of north and central Malaita, Solomon Islands: the thickest and most accessible part of the world's largest (Ontong Java) ocean plateau. *Geol. Soc. London, Spec. Publ.* **229**, 63–81 (2004).
51. Christensen, N. I. Poisson's ratio and crustal seismology. *J. Geophys. Res. Solid Earth* **101**, 3139–3156 (1996).
52. Coffin, M. F. & Eldholm, O. Large igneous provinces: Crustal structure, dimensions, and external consequences. *Rev. Geophys.* **32**, 1 (1994).
53. Fitton, J. G. & Godard, M. Origin and evolution of magmas on the Ontong Java Plateau. *Geol. Soc. London, Spec. Publ.* **229**, 151–178 (2004).
54. Tejada, M. L. G. Basement Geochemistry and Geochronology of Central Malaita, Solomon Islands, with Implications for the Origin and Evolution of the Ontong Java Plateau. *J. Petrol.* **43**, 449–484 (2002).
55. Reekie, C. D. J. *et al.* Sulfide resorption during crustal ascent and degassing of oceanic plateau basalts. *Nat. Commun.* **10**, 82 (2019).
56. Karlstrom, L. & Richards, M. On the evolution of large ultramafic magma chambers and timescales for flood basalt eruptions. *J. Geophys. Res.* **116**, B08216 (2011).
57. Tharimena, S., Rychert, C. A. & Harmon, N. Seismic imaging of a mid-lithospheric discontinuity beneath Ontong Java Plateau. *Earth Planet. Sci. Lett.* **450**, 62–70 (2016).
58. Tonegawa, T. *et al.* Characterization of Crustal and Uppermost-Mantle Seismic Discontinuities in the Ontong Java Plateau. *J. Geophys. Res. Solid Earth* **124**, 7155–7170 (2019).
59. Roman, A. & Arndt, N. Differentiated Archean oceanic crust: Its thermal structure, mechanical stability and a test of the sagduction hypothesis. *Geochim. Cosmochim. Acta* **278**, 65–77 (2020).
60. Hochmuth, K., Gohl, K., Uenzelmann-Neben, G. & Werner, R. Multiphase magmatic and tectonic evolution of a large igneous province - Evidence from the crustal structure of the Manihiki Plateau, western Pacific. *Tectonophysics* **750**, 434–457 (2019).
61. Ágústsson, K. & Flóvenz, Ó. G. The thickness of the seismogenic crust in Iceland and its

implications for geothermal systems. in *Proceedings of the World Geothermal Congress* 24–29 (2005).

62. Björnsson, A. Temperature of the Icelandic crust: Inferred from electrical conductivity, temperature surface gradient, and maximum depth of earthquakes. *Tectonophysics* **447**, 136–141 (2008).

63. Molnar, P. The Brittle-Plastic Transition, Earthquakes, Temperatures, and Strain Rates. *J. Geophys. Res. Solid Earth* **125**, (2020).

64. Mutch, E. J. F., Maclennan, J., Shorttle, O., Edmonds, M. & Rudge, J. F. Rapid transcrustal magma movement under Iceland. *Nat. Geosci.* **12**, 569–574 (2019).

65. Jenkins, J. *et al.* Crustal Formation on a Spreading Ridge Above a Mantle Plume: Receiver Function Imaging of the Icelandic Crust. *J. Geophys. Res. Solid Earth* **123**, 5190–5208 (2018).

66. Nixon, P. H. & Boyd, F. R. Garnet bearing lherzolites and discrete nodule suites from the Malaita Alnoite, Solomon Islands, S.W. Pacific, and their bearing on oceanic mantle composition and geotherm. in *The Mantle Sample: Inclusion in Kimberlites and Other Volcanics* 400–423 (American Geophysical Union, 1979). doi:10.1029/SP016p0400.

67. Ishikawa, A. Layered Lithospheric Mantle Beneath the Ontong Java Plateau: Implications from Xenoliths in Alnoite, Malaita, Solomon Islands. *J. Petrol.* **45**, 2011–2044 (2004).

68. Ishikawa, A., Pearson, D. G. & Dale, C. W. Ancient Os isotope signatures from the Ontong Java Plateau lithosphere: Tracing lithospheric accretion history. *Earth Planet. Sci. Lett.* **301**, 159–170 (2011).

69. Bédard, J. H. Stagnant lids and mantle overturns: Implications for Archaean tectonics, magmagenesis, crustal growth, mantle evolution, and the start of plate tectonics. *Geosci. Front.* **9**, 19–49 (2018).

70. Hawkesworth, C. J., Cawood, P. A. & Dhuime, B. The Evolution of the Continental Crust and the Onset of Plate Tectonics. *Front. Earth Sci.* **8**, (2020).

71. Weller, O. M., Copley, A., Miller, W. G. R., Palin, R. M. & Dyck, B. The relationship between mantle potential temperature and oceanic lithosphere buoyancy. *Earth Planet. Sci. Lett.* **518**, 86–99 (2019).

72. van Hunen, J. & Moyen, J.-F. Archean Subduction: Fact or Fiction? *Annu. Rev. Earth Planet. Sci.* **40**, 195–219 (2012).

73. Chowdhury, P., Chakraborty, S., Gerya, T. V., Cawood, P. A. & Capitanio, F. A. Peel-back controlled lithospheric convergence explains the secular transitions in Archean metamorphism and magmatism. *Earth Planet. Sci. Lett.* **538**, 116224 (2020).
74. Lourenço, D. L. & Rudolph, M. L. Shallow Lower Mantle Viscosity Modulates the Pattern of Mantle Structure. *Geochemistry, Geophys. Geosystems* **21**, (2020).
75. Lin, S., Parks, J., Heaman, L. M., Simonetti, A. & Corkery, M. T. Diapirism and sagduction as a mechanism for deposition and burial of "Timiskaming-type" sedimentary sequences, Superior Province: Evidence from detrital zircon geochronology and implications for the Borden Lake conglomerate in the exposed middle to lower. *Precambrian Res.* **238**, 148–157 (2013).
76. Hao, L.-L. *et al.* Oceanic plateau subduction during closure of the Bangong-Nujiang Tethyan Ocean: Insights from central Tibetan volcanic rocks. *GSA Bull.* **131**, 864–880 (2019).
77. Zhang, L., Li, S. & Zhao, Q. A review of research on adakites. *Int. Geol. Rev.* 1–18 (2019) doi:10.1080/00206814.2019.1702592.
78. Wang, Q. *et al.* Ridge subduction, magmatism, and metallogenesis. *Sci. China Earth Sci.* (2020) doi:10.1007/s11430-019-9619-9.
79. Kay, S. M., Ramos, V. A. & Marquez, M. Evidence in Cerro Pampa Volcanic Rocks for Slab-Melting Prior to Ridge-Trench Collision in Southern South America. *J. Geol.* **101**, 703–714 (1993).
80. Király, Á. *et al.* The effect of slab gaps on subduction dynamics and mantle upwelling. *Tectonophysics* **785**, 228458 (2020).
81. Jadamec, M. A. Insights on slab-driven mantle flow from advances in three-dimensional modelling. *J. Geodyn.* **100**, 51–70 (2016).
82. Berkelhammer, S. E. *et al.* Geochemical and geochronological records of tectonic changes along a flat-slab arc-transform junction: Circa 30 Ma to ca. 19 Ma Sonya Creek volcanic field, Wrangell Arc, Alaska. *Geosphere* **15**, 1508–1538 (2019).
83. Li, S.-M. *et al.* Slab-derived adakites and subslab asthenosphere-derived OIB-type rocks at 156 ± 2 Ma from the north of Gerze, central Tibet: Records of the Bangong–Nujiang oceanic ridge subduction during the Late Jurassic. *Lithos* **262**, 456–469 (2016).
84. Wu, C., Zheng, Y., Xu, B. & Hou, Z. The genetic relationship between JTA–like magmas

and typical adakites: An example from the Late Cretaceous Nuri complex, southern Tibet. *Lithos* **320**–**321**, 265–279 (2018).

85. Halla, J. The TTG-Amphibolite Terrains of Arctic Fennoscandia: Infinite Networks of Amphibolite Metatexite-Diatexite Transitions. *Front. Earth Sci.* **8**, 1–14 (2020).

86. Breitfeld, H. T. *et al.* Adakites without a slab: Remelting of hydrous basalt in the crust and shallow mantle of Borneo to produce the Miocene Sintang Suite and Bau Suite magmatism of West Sarawak. *Lithos* **344**–**345**, 100–121 (2019).

87. Koloskov, A. V., Kovalenko, D. V. & Ananyev, V. V. Adakite Volcanism at Continental Margin and Associated Problems. Part II. Adakites from the Sea of Okhotsk, Kamchatka, and Bering Sea Regions: Typification and Genesis. *Russ. J. Pacific Geol.* **13**, 417–435 (2019).

88. Kay, S. M. *et al.* The Calc-Alkaline Hidden Bay and Kagalaska Plutons and the Construction of the Central Aleutian Oceanic Arc Crust. *J. Petrol.* **60**, 393–439 (2019).

89. Jicha, B. R. & Kay, S. M. Quantifying arc migration and the role of forearc subduction erosion in the central Aleutians. *J. Volcanol. Geotherm. Res.* **360**, 84–99 (2018).

90. Mahar, M. A., Pavlis, T. L., Bowman, J. R., Conrad, W. K. & Goodell, P. C. Early Cretaceous ridge subduction beneath southern Alaska: Insights from zircon U-Pb geochronology, hafnium, and oxygen isotopic compositions of the Western Chugach tonalite-trondhjemite suite. *GSA Bull.* **131**, 521–546 (2019).

91. Zeng, Y.-C., Xu, J.-F., Huang, F., Li, M.-J. & Chen, Q. Generation of the 105–100 Ma Dagze volcanic rocks in the north Lhasa Terrane by lower crustal melting at different temperature and depth: Implications for tectonic transition. *GSA Bull.* **132**, 1257–1272 (2020).

92. André, L. *et al.* Early continental crust generated by reworking of basalts variably silicified by seawater. *Nat. Geosci.* **12**, 769–773 (2019).

93. Schnetzler, C. . & Philpotts, J. A. Partition coefficients of rare-earth elements between igneous matrix material and rock-forming mineral phenocrysts—II. *Geochim. Cosmochim. Acta* **34**, 331–340 (1970).

94. Dudas, M. J., Schmitt, R. A. & Harward, M. E. Trace element partitioning between volcanic plagioclase and dacitic pyroclastic matrix. *Earth Planet. Sci. Lett.* **11**, 440–446 (1971).

95. Nagasawa, H. & Schnetzler, C. C. Partitioning of rare earth, alkali and alkaline earth elements between phenocrysts and acidic igneous magma. *Geochim. Cosmochim. Acta* **35**, 953–968 (1971).
96. Laurent, O. *et al.* Earth's earliest granitoids are crystal-rich magma reservoirs tapped by silicic eruptions. *Nat. Geosci.* **13**, 163–169 (2020).
97. Doucet, L. S. *et al.* Archean lithospheric differentiation: Insights from Fe and Zn isotopes. *Geology* **XX**, 1–5 (2020).
98. Clarke, D. B. The Origins of Strongly Peraluminous Granitoid Rocks. *Can. Mineral.* **57**, 529–550 (2019).
99. Simon, L. & Lécuyer, C. Continental recycling: The oxygen isotope point of view. *Geochemistry, Geophys. Geosystems* **6**, (2005).
100. Huang, S., Farkaš, J. & Jacobsen, S. B. Stable calcium isotopic compositions of Hawaiian shield lavas: Evidence for recycling of ancient marine carbonates into the mantle. *Geochim. Cosmochim. Acta* **75**, 4987–4997 (2011).
101. Amsellem, E. *et al.* Calcium isotopic evidence for the mantle sources of carbonatites. *Sci. Adv.* **6**, (2020).
102. Chen, C. *et al.* Calcium isotope evidence for subduction-enriched lithospheric mantle under the northern North China Craton. *Geochim. Cosmochim. Acta* **238**, 55–67 (2018).
103. Kang, J. T. *et al.* Calcium isotopic fractionation in mantle peridotites by melting and metasomatism and Ca isotope composition of the Bulk Silicate Earth. *Earth Planet. Sci. Lett.* **474**, 128–137 (2017).
104. Liu, F. *et al.* Marine Carbonate Component in the Mantle Beneath the Southeastern Tibetan Plateau: Evidence From Magnesium and Calcium Isotopes. *J. Geophys. Res. Solid Earth* **122**, 9729–9744 (2017).
105. Kang, J. T. *et al.* Calcium isotopic composition of mantle xenoliths and minerals from Eastern China. *Geochim. Cosmochim. Acta* **174**, 335–344 (2016).
106. Huang, S., Farkaš, J. & Jacobsen, S. B. Calcium isotopic fractionation between clinopyroxene and orthopyroxene from mantle peridotites. *Earth Planet. Sci. Lett.* **292**, 337–344 (2010).
107. Banerjee, A. & Chakrabarti, R. A geochemical and Nd, Sr and stable Ca isotopic study of carbonatites and associated silicate rocks from the ~65 Ma old Ambadongar carbonatite

complex and the Phenai Mata igneous complex, Gujarat, India. *LITHOS* **326–327**, 572–585 (2019).

108. Fantle, M. S. & Tipper, E. T. Calcium isotopes in the global biogeochemical Ca cycle: Implications for development of a Ca isotope proxy. *Earth-Science Rev.* **129**, 148–177 (2014).

109. Blättler, C. L. & Higgins, J. A. Testing Urey's carbonate–silicate cycle using the calcium isotopic composition of sedimentary carbonates. *Earth Planet. Sci. Lett.* **479**, 241–251 (2017).

110. Ionov, D. A. *et al.* Calcium isotopic signatures of carbonatite and silicate metasomatism, melt percolation and crustal recycling in the lithospheric mantle. *Geochim. Cosmochim. Acta* **248**, 1–13 (2019).

111. Holmden, C., Papanastassiou, D. a., Blanchon, P. & Evans, S. δ 44/40Ca variability in shallow water carbonates and the impact of submarine groundwater discharge on Ca-cycling in marine environments. *Geochim. Cosmochim. Acta* **83**, 179–194 (2012).

112. Dauphas, N., Cates, N. L., Mojzsis, S. J. & Busigny, V. Identification of chemical sedimentary protoliths using iron isotopes in the > 3750 Ma Nuvvuagittuq supracrustal belt, Canada. *Earth Planet. Sci. Lett.* **254**, 358–376 (2007).

113. Boehnke, P. *et al.* Potassic, high-silica Hadean crust. *Proc. Natl. Acad. Sci.* **115**, 6353–6356 (2018).

114. Chowdhury, W. *et al.* Geochemical and textural investigations of the Eoarchean Ukaliq supracrustals, Northern Québec (Canada). *Lithos* **372–373**, 105673 (2020).

115. Cates, N. L. & Mojzsis, S. J. Metamorphic zircon, trace elements and Neoarchean metamorphism in the ca. 3.75 Ga Nuvvuagittuq supracrustal belt, Québec (Canada). *Chem. Geol.* **261**, 99–114 (2009).

116. Cates, N. L. & Mojzsis, S. J. Pre-3750 Ma supracrustal rocks from the Nuvvuagittuq supracrustal belt, northern Québec. *Earth Planet. Sci. Lett.* **255**, 9–21 (2007).

117. Greer, J. *et al.* Widespread poly-metamorphosed Archean granitoid gneisses and supracrustal enclaves of the southern Inukjuak Domain, Québec (Canada). *Lithos* **364–365**, 105520 (2020).

118. Mloszewska, A. M. *et al.* The composition of Earth's oldest iron formations: The Nuvvuagittuq Supracrustal Belt (Québec, Canada). *Earth Planet. Sci. Lett.* **317–318**, 331–

342 (2012).

119. Mloszewska, A. M. *et al.* Chemical sedimentary protoliths in the >3.75Ga Nuvvuagittuq Supracrustal Belt (Québec, Canada). *Gondwana Res.* **23**, 574–594 (2013).

120. Adam, J., Rushmer, T., O'Neil, J. & Francis, D. Hadean greenstones from the Nuvvuagittuq fold belt and the origin of the Earth's early continental crust. *Geology* **40**, 363–366 (2012).

121. Farkaš, J. *et al.* Calcium isotope record of Phanerozoic oceans: Implications for chemical evolution of seawater and its causative mechanisms. *Geochim. Cosmochim. Acta* **71**, 5117–5134 (2007).

122. Putirka, K. Rates and styles of planetary cooling on Earth, Moon, Mars, and Vesta, using new models for oxygen fugacity, ferric-ferrous ratios, olivine-liquid Fe-Mg exchange, and mantle potential temperature. *Am. Mineral.* **101**, 819–840 (2016).

123. Ganne, J. & Feng, X. Primary magmas and mantle temperatures through time. *Geochemistry, Geophys. Geosystems* **18**, 872–888 (2017).

124. Amini, M. *et al.* Calcium isotope (δ44/40Ca) fractionation along hydrothermal pathways, Logatchev field (Mid-Atlantic Ridge, 14 45 N). *Geochim. Cosmochim. Acta* **72**, 4107–4122 (2008).

125. Scheuermann, P. P., Syverson, D. D., Higgins, J. A., Pester, N. J. & Seyfried, W. E. Calcium isotope systematics at hydrothermal conditions: Mid-ocean ridge vent fluids and experiments in the CaSO4-NaCl-H2O system. *Geochim. Cosmochim. Acta* **226**, 18–35 (2018).

126. Schmitt, A. D., Chabaux, F. & Stille, P. The calcium riverine and hydrothermal isotopic fluxes and the oceanic calcium mass balance. *Earth Planet. Sci. Lett.* **213**, 503–518 (2003).

127. Antonelli, M. A., Depaolo, D. J., Brown, S. T. & Pester, N. J. Radiogenic 40Ca in Seawater. *Goldschmidt Conf. 2018, Boston, MA* (2018).

128. German, C. R. & Seyfried, W. E. *Hydrothermal Processes*. *The Oceans and Marine Geochemistry* vol. 8 (Elsevier Ltd., 2014).

129. Antonelli, M. A., DePaolo, D. J., Chacko, T., Grew, E. S. & Rubatto, D. Radiogenic Ca isotopes confirm post-formation K depletion of lower crust. *Geochemical Perspect. Lett.* **9**, 43–48 (2019).

130. Veizer, J., Compston, W., Hoefs, J. & Nielsen, H. Mantle buffering of the early oceans. *Naturwissenschaften* **69**, 173–180 (1982).
131. Kump, L. R. & Seyfried, W. E. Hydrothermal Fe fluxes during the Precambrian: Effect of low oceanic sulfate concentrations and low hydrostatic pressure on the composition of black smokers. *Earth Planet. Sci. Lett.* **235**, 654–662 (2005).
132. Djokic, T., Van Kranendonk, M. J., Campbell, K. A., Walter, M. R. & Ward, C. R. Earliest signs of life on land preserved in ca. 3.5 Ga hot spring deposits. *Nat. Commun.* **8**, 15263 (2017).
133. Van Kranendonk, M. Volcanic degassing, hydrothermal circulation and the flourishing of early life on Earth: A review of the evidence from c. 3490-3240 Ma rocks of the Pilbara Supergroup, Pilbara Craton, Western Australia. *Earth-Science Rev.* **74**, 197–240 (2006).
134. Nutman, A. P., Friend, C. R. L., Bennett, V. C., Wright, D. & Norman, M. D. ≥3700Ma pre-metamorphic dolomite formed by microbial mediation in the Isua supracrustal belt (W. Greenland): Simple evidence for early life? *Precambrian Res.* **183**, 725–737 (2010).
135. Nutman, A. P., Bennett, V. C., Friend, C. R. L., Van Kranendonk, M. J. & Chivas, A. R. Rapid emergence of life shown by discovery of 3,700-million-year-old microbial structures. *Nature* **537**, 535–538 (2016).
136. Nutman, A. P. *et al.* Cross-examining Earth's oldest stromatolites: Seeing through the effects of heterogeneous deformation, metamorphism and metasomatism affecting Isua (Greenland) ∼3700 Ma sedimentary rocks. *Precambrian Res.* **331**, 105347 (2019).
137. Grotzinger, J. P. & James, N. P. Precambrian carbonates: evolution and understanding. in *Carbonate Sedimentation and Diagenesis in the Evolving Precambrian World* 3–20 (SEPM (Society for Sedimentary Geology), 2000). doi:10.2110/pec.00.67.0003.
138. Tang, J., Köhler, S. J. & Dietzel, M. Sr2+/Ca2+ and 44Ca/40Ca fractionation during inorganic calcite formation: I. Sr incorporation. *Geochim. Cosmochim. Acta* **72**, 3718–3732 (2008).
139. Gussone, N. *et al.* Calcium isotope fractionation in calcite and aragonite. *Geochim. Cosmochim. Acta* **69**, 4485–4494 (2005).
140. Wilde, S. A., Valley, J. W., Peck, W. H. & Graham, C. M. Evidence from detrital zircons for the existence of continental crust and oceans on the Earth 4.4 Gyr ago. *Nature* **409**, 175–178 (2001).

141. Mojzsis, S. J., Harrison, T. M. & Pidgeon, R. T. Oxygen-isotope evidence from ancient zircons for liquid water at the Earth's surface 4,300 Myr ago. *Nature* **409**, 178–181 (2001).
142. Charnay, B., Wolf, E. T., Marty, B. & Forget, F. Is the Faint Young Sun Problem for Earth Solved? *Space Sci. Rev.* **216**, 90 (2020).
143. Hart, M. H. The evolution of the atmosphere of the earth. *Icarus* **33**, 23–39 (1978).
144. Martinez, M. P. & Turi, B. The isotopic composition of oxygen and carbon in the hyaloclastites from the Mt. Iblei volcanic area, Eastern Sicily: A preliminary study. *Bull. Volcanol.* **41**, 168–174 (1978).
145. Kemp, A. I. S. *et al.* Magmatic and Crustal Differentiation History of Granitic Rocks from Hf-O Isotopes in Zircon. *Science (80-.).* **315**, 980–983 (2007).
146. Yakymchuk, C. *et al.* Paleozoic evolution of western Marie Byrd Land, Antarctica. *Geol. Soc. Am. Bull.* **127**, 1464–1484 (2015).
147. Glazner, A. F. Thermal limitations on incorporation of wall rock into magma. *Geology* **35**, 319 (2007).
148. Méheut, M. & Schauble, E. A. Silicon isotope fractionation in silicate minerals: Insights from first-principles models of phyllosilicates, albite and pyrope. *Geochim. Cosmochim. Acta* **134**, 137–154 (2014).
149. Qin, T., Wu, F., Wu, Z. & Huang, F. First-principles calculations of equilibrium fractionation of O and Si isotopes in quartz, albite, anorthite, and zircon. *Contrib. to Mineral. Petrol.* **171**, 1–14 (2016).
150. Li, Y., Wang, W., Zhou, C. & Huang, F. First-principles calculations of equilibrium silicon isotope fractionation in metamorphic silicate minerals. *Solid Earth Sci.* **4**, 142–149 (2019).
151. Li, Y., Yu, H., Gu, X., Guo, S. & Huang, F. Silicon isotopic fractionation during metamorphic fluid activities: constraints from eclogites and ultrahigh-pressure veins in the Dabie orogen, China. *Chem. Geol.* **540**, 119550 (2020).
152. Malviya, S. *et al.* Insights into global diatom distribution and diversity in the world's ocean. *Proc. Natl. Acad. Sci. U. S. A.* **113**, E1516–E1525 (2016).
153. Wakita, K. Tectonic setting required for the preservation of sedimentary mélanges in Palaeozoic and Mesozoic accretionary complexes of southwest Japan. *Gondwana Res.* **74**,

90–100 (2019).

154. Shervais, J. W. The significance of subduction-related accretionary complexes in early Earth processes. *Spec. Pap. Geol. Soc. Am.* **405**, 173–192 (2006).

155. Turner, S., Rushmer, T., Reagan, M. & Moyen, J.-F. Heading down early on? Start of subduction on Earth. *Geology* **42**, 139–142 (2014).

156. Cawood, P. A. *et al.* Accretionary orogens through Earth history. *Geol. Soc. London, Spec. Publ.* **318**, 1–36 (2009).

157. Isozaki, Y., Aoki, K., Nakama, T. & Yanai, S. New insight into a subduction-related orogen: A reappraisal of the geotectonic framework and evolution of the Japanese Islands. *Gondwana Res.* **18**, 82–105 (2010).

158. Brown, M. & Johnson, T. *Global age, temperature and pressure data for secular change in metamorphism, Version 1.0. Interdisciplinary Earth Data Alliance (IEDA)*. (2019) doi:https://doi.org/10.1594/IEDA/111316.

159. Brown, M., Kirkland, C. L. & Johnson, T. E. Evolution of geodynamics since the Archean: Significant change at the dawn of the Phanerozoic. *Geology* **48**, 488–492 (2020).

160. Cates, N. L., Ziegler, K., Schmitt, A. K. & Mojzsis, S. J. Reduced, reused and recycled: Detrital zircons define a maximum age for the Eoarchean (ca. 3750-3780Ma) Nuvvuagittuq Supracrustal Belt, Québec (Canada). *Earth Planet. Sci. Lett.* **362**, 283–293 (2013).

161. Bibikova, E. V., Turkina, O. M., Kirnozova, T. I. & Fugzan, M. M. Ancient plagiogneisses of the Onot block of the Sharyzhalgai metamorphic massif: Isotopic geochronology. *Geochemistry Int.* **44**, 310–315 (2006).

162. Turkina, O. M. *et al.* Paleoarchean tonalite-trondhjemite complex in the northwestern part of the Sharyzhalgai uplift (southwestern Siberian craton): results of U-Pb and Sm-Nd study. *Russ. Geol. Geophys.* **50**, 15–28 (2009).

163. Cates, N. L. & Mojzsis, S. J. Chemical and isotopic evidence for widespread Eoarchean metasedimentary enclaves in southern West Greenland. *Geochim. Cosmochim. Acta* **70**, 4229–4257 (2006).

164. Martin, H. Petrogenesis of archaean trondhjemites, tonalites, and granodiorites from Eastern Finland: Major and trace element geochemistry. *J. Petrol.* **28**, 921–953 (1987).

165. Sanchez-Garrido, C. J. M. G. *et al.* Diversity in Earth's early felsic crust: Paleoarchean

peraluminous granites of the Barberton Greenstone Belt. *Geology* **39**, 963–966 (2011).

III. Supplementary Figures

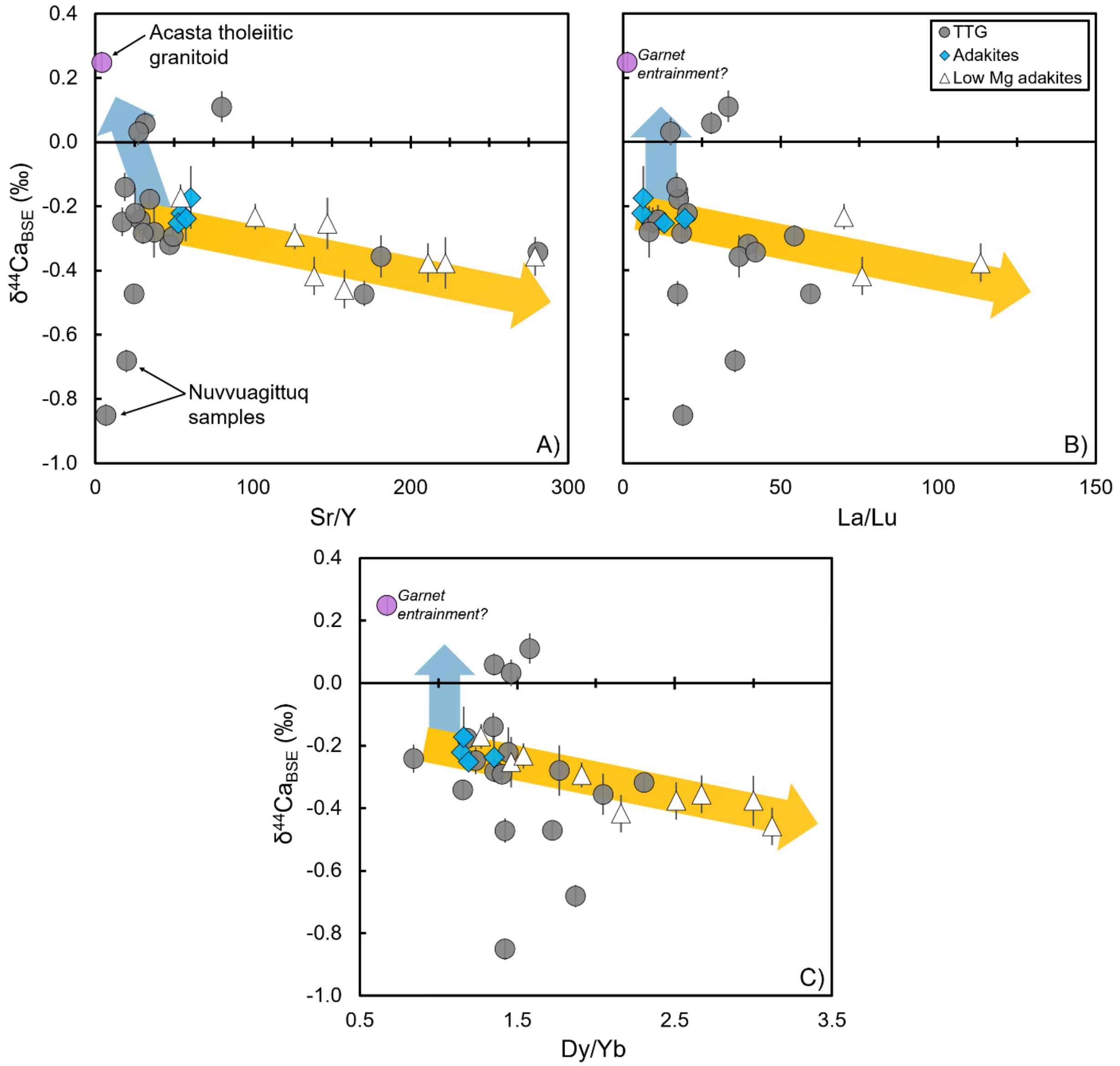

Supplementary Fig. 1.

Ca isotope compositions *vs.* trace-element ratios indicative of residual garnet in TTG and adakite source rocks. Arrows represent estimated trends for garnet fractionation (orange) and plagioclase fractionation (blue). (A) δ^{44}Ca *vs.* Sr/Y. (B) δ^{44}Ca *vs.* Dy/Yb. (C) δ^{44}Ca *vs.* La/Lu. Low-Mg adakite data[29] are shown for comparison. Entrainment of garnet observed in the Acasta tholeiitic granitoid (pink circle) can account for its high δ^{44}Ca and low Sr/Y, La/Lu, and Dy/Yb. Error bars represent 2SE uncertainties on Ca isotope measurements. Tonalite-trondjhemite-granodiorite suite (‘TTG’).

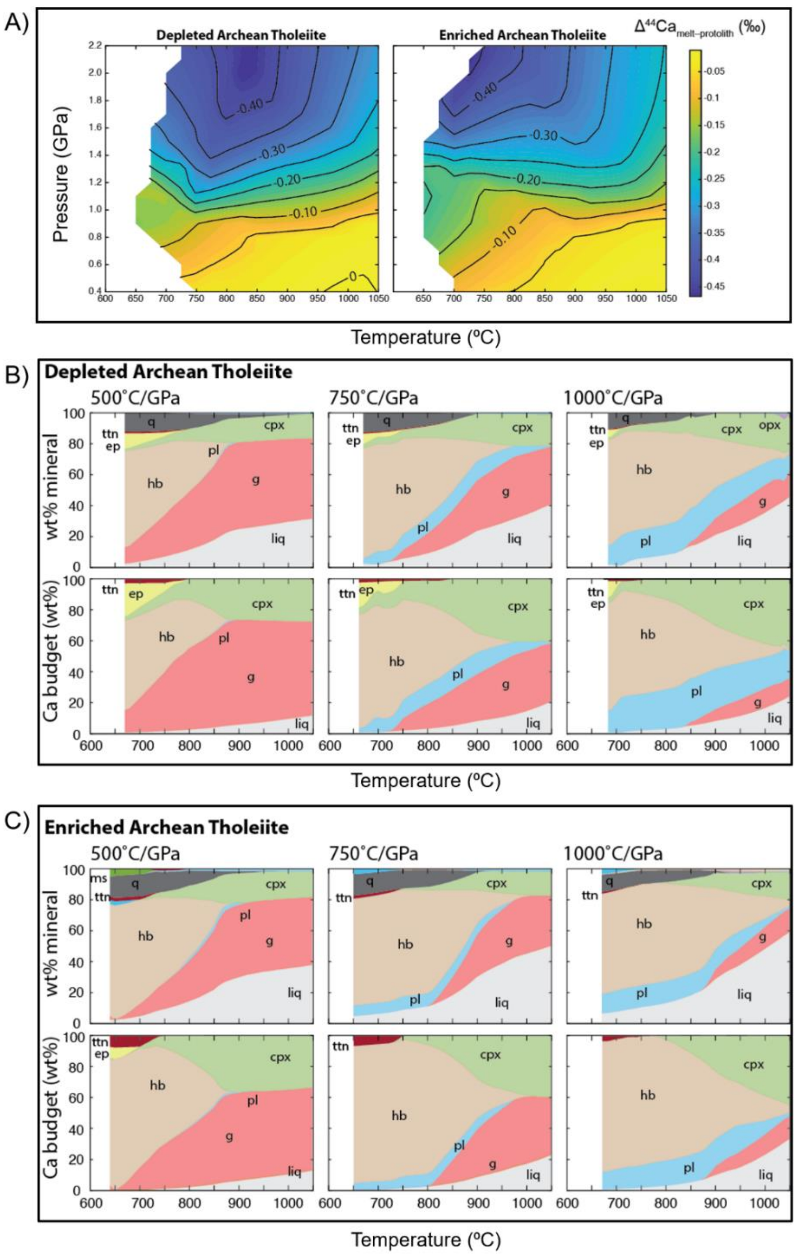

Supplementary Fig. 2.

(A) Calcium isotope contour plots in pressure-temperature space for depleted Archean tholeiite (left) and enriched Archean tholeiite (right). These plots show the general competition between pressure and temperature but cannot be directly compared to the other model results because they are grid-calculations and do not consider evolution along different geothermal gradients. (B, C) Evolution of residual mineral assemblages and Ca budgets (considering proportions and Ca concentrations of each mineral) with increasing temperatures, from closed system phase equilibrium models along three different geothermal gradients (500, 750, & 1000ºC/GPa); (B) Depleted Archean tholeiite protolith, (C) Enriched Archean tholeiite protolith. Muscovite (‘ms’), quartz (‘q’), titanite (‘ttn’), epidote (‘ep’), hornblende (‘hb’), plagioclase (‘pl’), clinopyroxene (‘cpx’), garnet (‘g’), liquid (‘liq’).

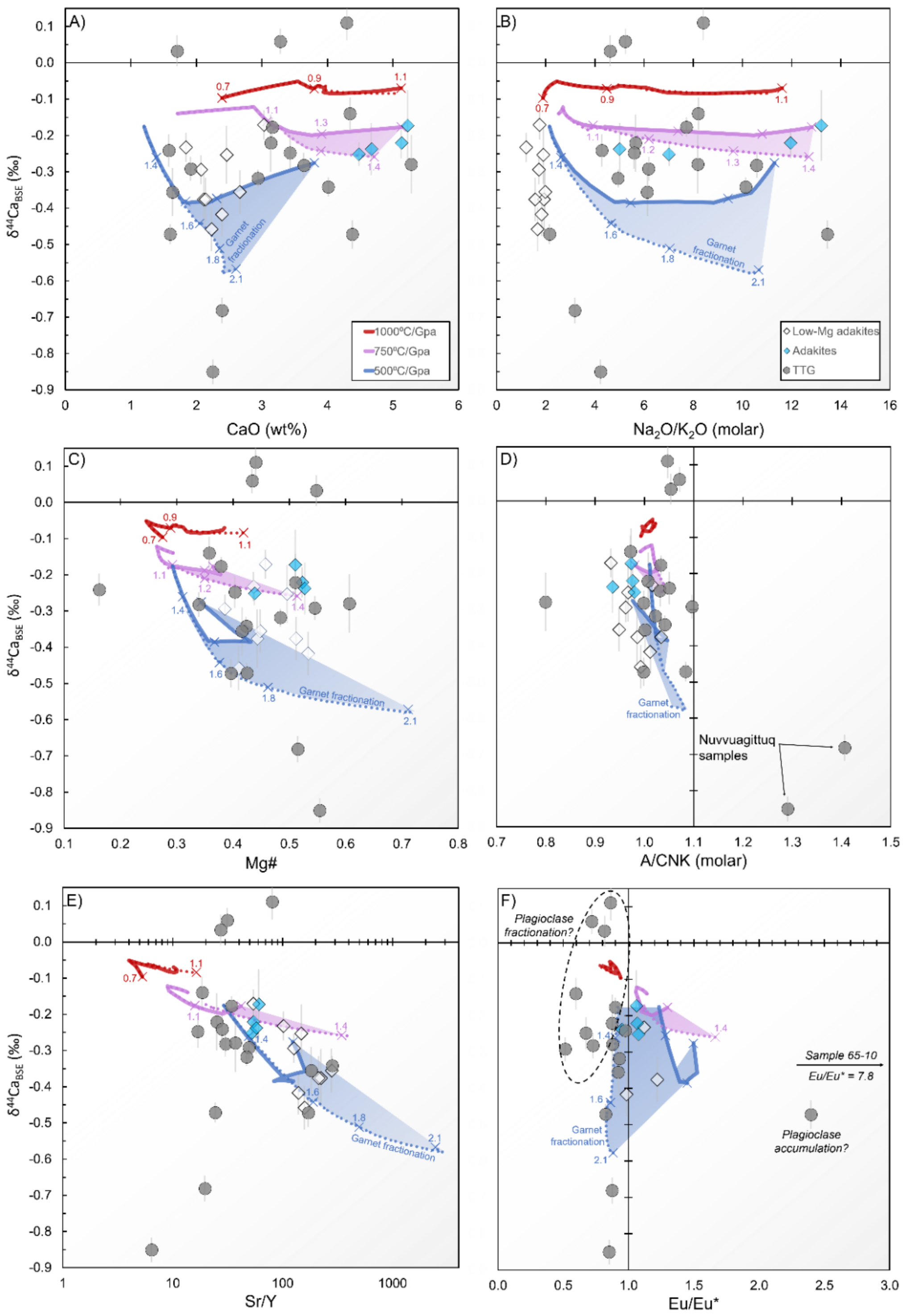

Supplementary Fig. 3.

Ca isotope compositions *vs.* major and trace element ratios for depleted Archean tholeiite (DAT) phase equilibrium modeling results and TTG/adakite data. Low-Mg adakite data[29] are shown for comparison. Error bars represent 2SE uncertainties on Ca isotope measurements. Tonalite-trondjhemite-granodiorite suite ('TTG').

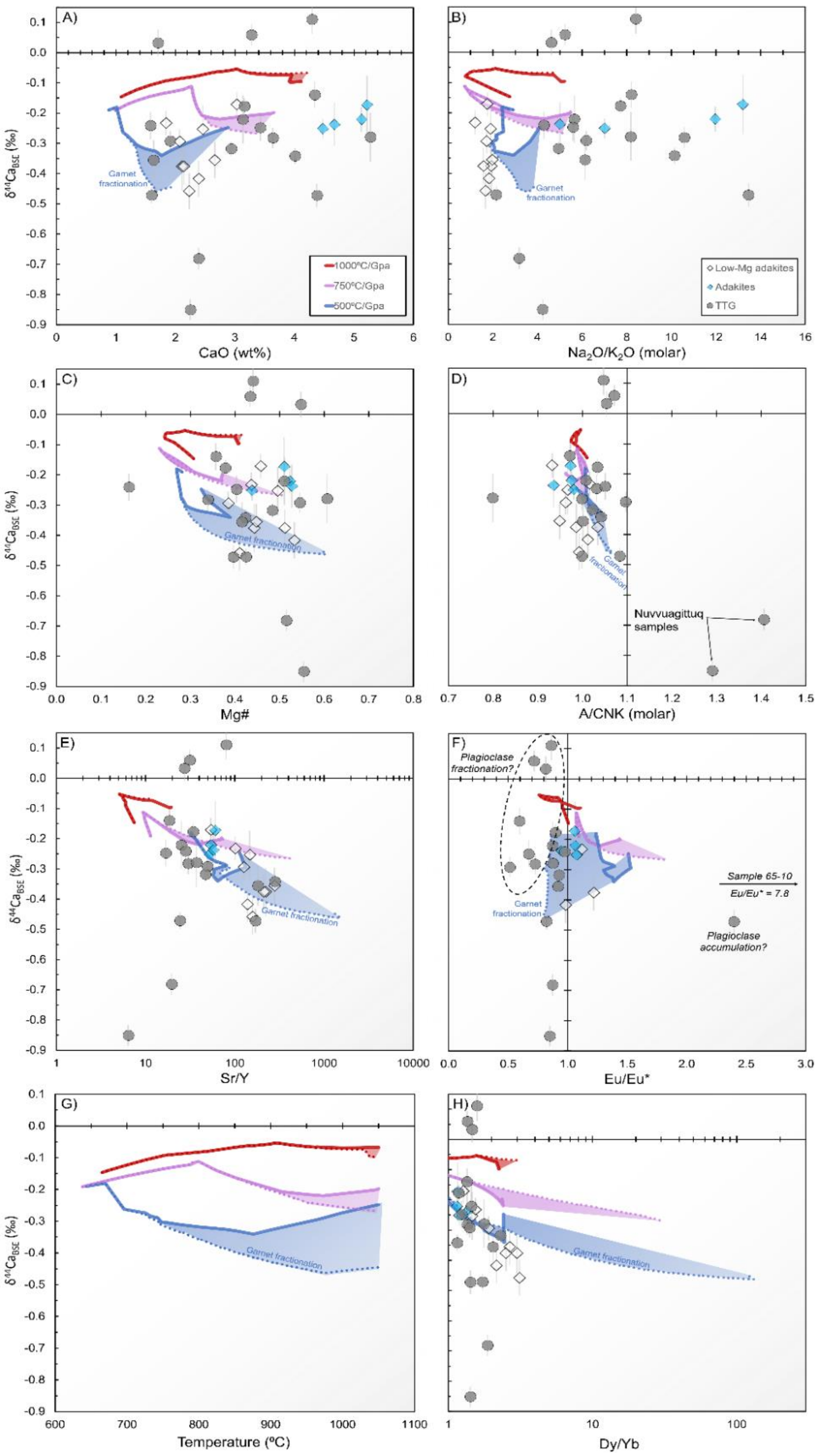

Supplementary Fig. 4.

Ca isotope compositions *vs.* major and trace element ratios for enriched Archean tholeiite (EAT) phase equilibrium modelling results and TTG/adakite data. Low-Mg adakite data[29] are shown for comparison. Error bars represent 2SE uncertainties on Ca isotope measurements. Tonalite-trondjhemite-granodiorite suite ('TTG').

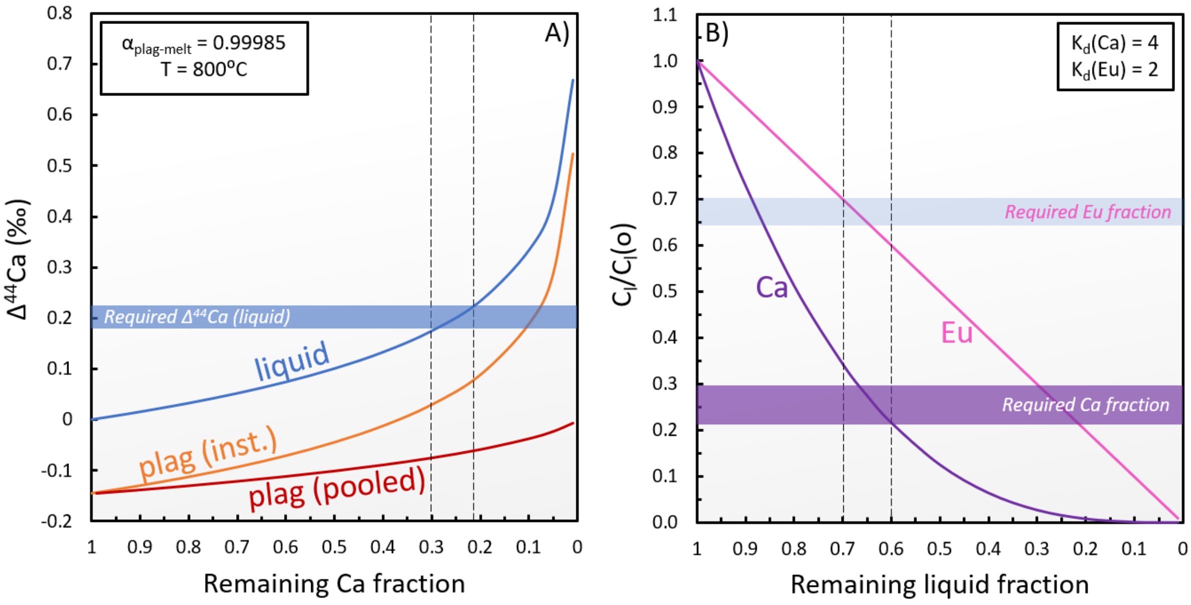

Supplementary Fig. 5.

Rayleigh fractionation and fractional crystallization models for plagioclase, required to explain TTG samples with δ^{44}Ca > 0‰ (n = 3). Assuming the parental melts had δ^{44}Ca -0.1‰, as would be the case for TTG melts produced at high geothermal gradients (1000ºC/GPa), a Δ^{44}Ca of +0.2‰ is required to explain the sample compositions (δ^{44}Ca up to +0.1‰) which corresponds to a remaining Ca fraction in the melt of ~0.25 (shown in panel A). Using average Eu and Ca distribution coefficients [K_d(Eu) and K_d(Ca), respectively] for plagioclase in dacitic melt[93–95] and assuming that the plagioclase-melt fractionation factor ($\alpha_{plag\text{-}melt}$) is 0.99985 and that initial melt has no Eu-anomaly, the Eu-anomalies and δ^{44}Ca values can be reproduced through ~30-40% fractional crystallization of plagioclase (shown in panel B). Plagioclase ('plag'), instantaneous ('inst.').

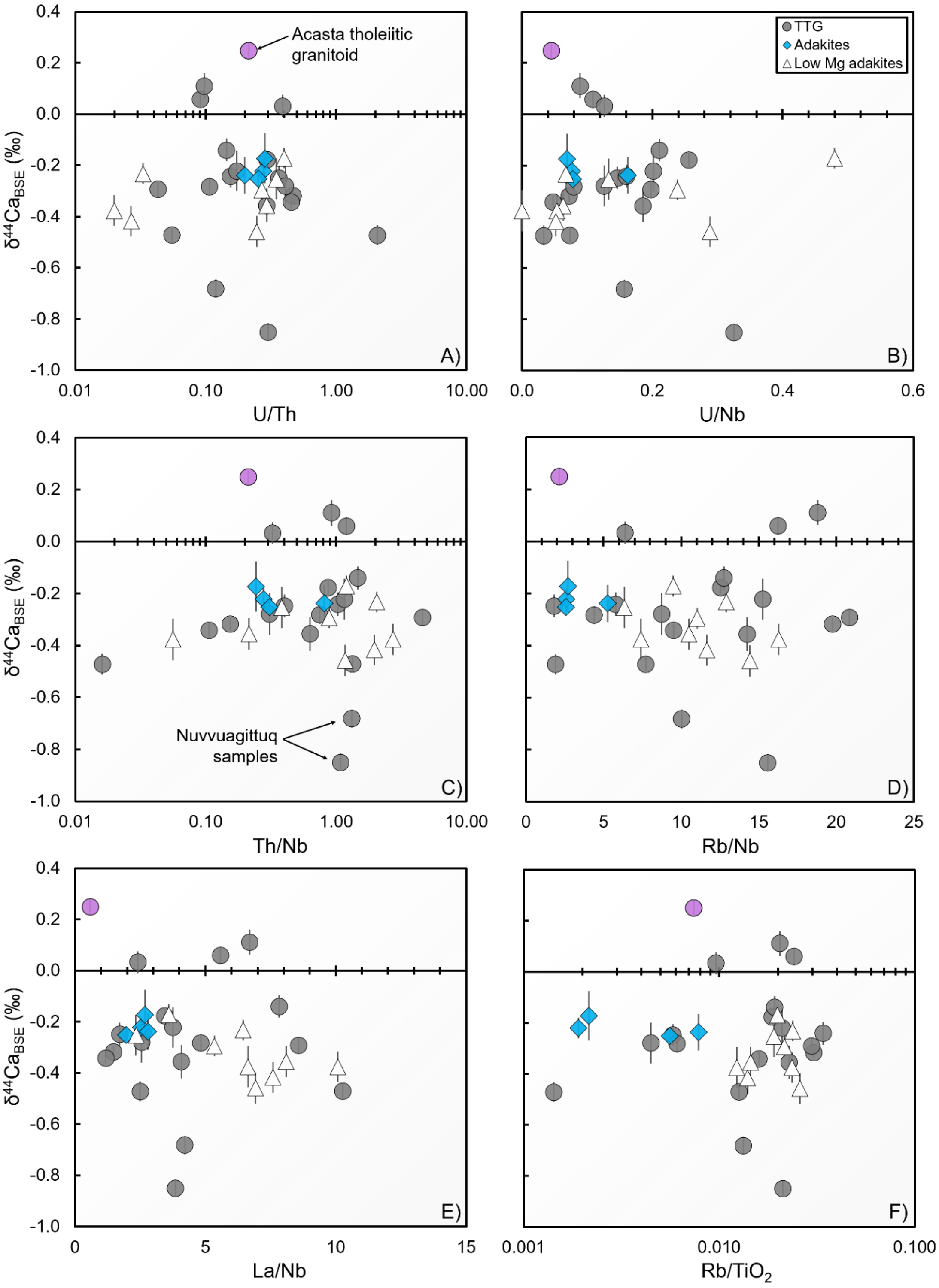

Supplementary Fig. 6.

Ca isotope compositions *vs.* fluid mobile/immobile trace elements measured by Q-ICP-MS. Low-Mg adakite data[29] are shown for comparison. Error bars represent 2SE uncertainties on Ca isotope measurements. Tonalite-trondjhemite-granodiorite suite ('TTG').

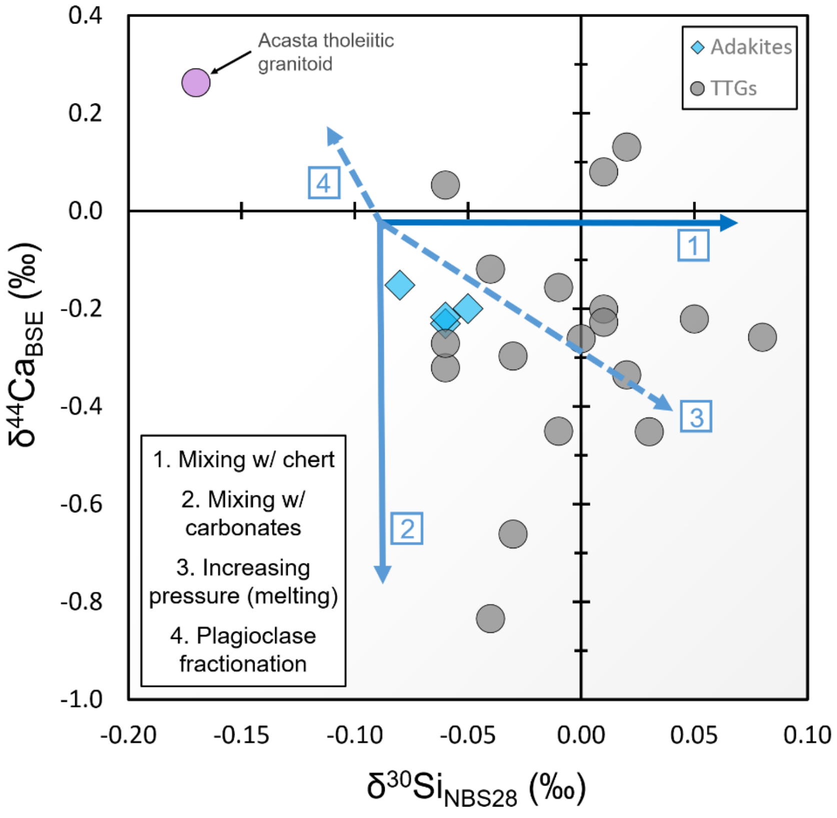

Supplementary Fig. 7.

Ca isotope *vs*. silicon isotope compositions[2] for adakite and Archean samples analyzed in this study. Solid blue arrows show the effects of incorporating low-T chert[2,92] and isotopically light Eoarchean carbonates (*this study*). Dotted blue arrows represent inferred trends based on the models in this study (for Ca isotopes) and the *ab-initio* and empirical predictions for equilibrium Si isotope partitioning, where garnet preferentially incorporates lighter Si isotopes and plagioclase incorporates heavier Si isotopes, relative to other major silicate phases/melt[148–151]. Tonalite-trondjhemite-granodiorite suite ('TTG'), with ('w/').

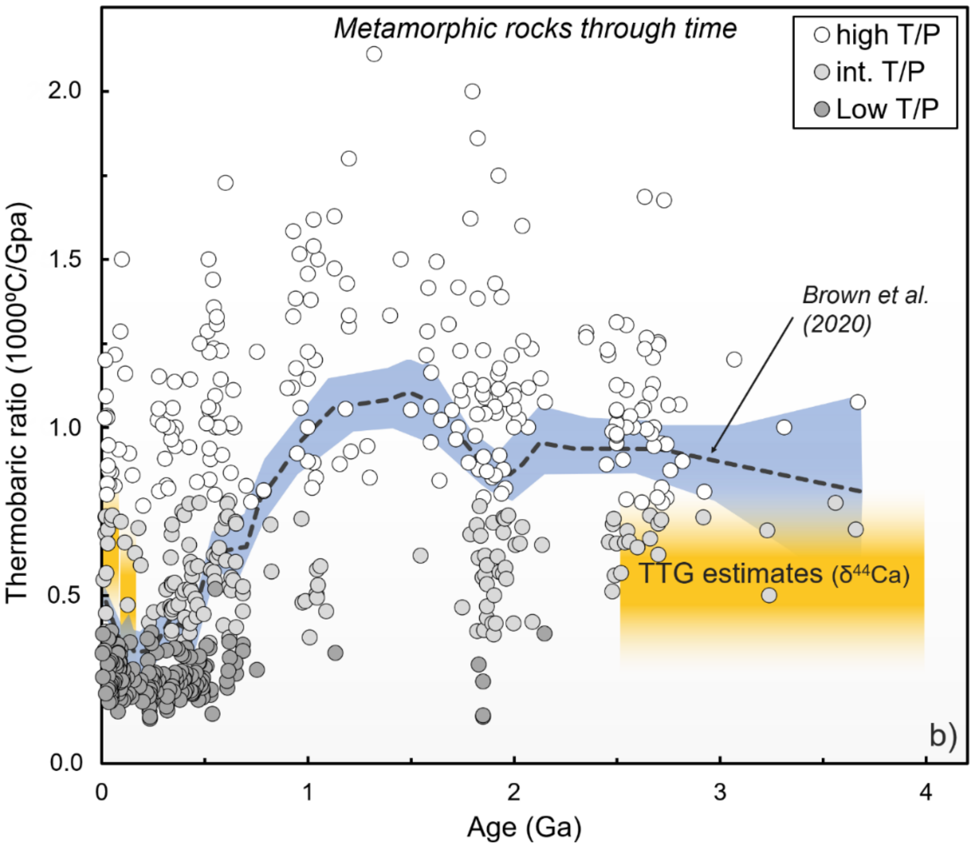

Supplementary Fig. 8.

Comparison of TTG estimates from this study (orange regions) to thermobaric ratio estimates for metamorphic rocks through geologic time. Data are from the dataset of Ref. 158. Averages through time (dotted black line with blue confidence intervals) are from Ref. 159. Tonalite-trondjhemite-granodiorite suite (‘TTG’), temperature/pressure (‘T/P’), intermediate (‘int.’).

IV. Supplementary Tables

Supplementary Table 1.

Isotopic data for the samples analyzed in this study.

Location	Type	Sample	Age (Ma)	δ^{44}Ca (BSE)	2SE	n	δ^{18}O (SMOW)	δ^{30}Si (NBS28)	εNd (t)	εHf (t)
Tungus-Anabar	TTG	28-03	3300	0.11	0.05	5	-	0.02	-	0.7
	TTG	40-03	3400	-0.18	0.03	5	-	-0.01	0.7	2.1
	TTG	58-03	3392	0.06	0.03	5	-	0.01	-	2.0
	TTG	60-04	3000	-0.28	0.03	5	-	0.00	-1.0	1.8
	TTG	65-10	2840	-0.34	0.03	5	-	-0.06	2.3	1.6
Baltic Shield	TTG	3-85	2850	-0.47	0.04	6	6.19	0.03	-	2.0
	TTG	74-2	2813	-0.14	0.04	5	-	-0.04	-	2.5
	TTG	H392	2809	-0.29	0.03	5	-	-0.06	2.0	3.3
	TTG	H395	2792	-0.22	0.08	5	-	0.01	2.0	2.9
Greenland	TTG	GRO4057	3692	-0.32	0.02	5	-	-0.03	-	1.2
Slave Craton	Tholeiite	AG09-008	3982	0.25	0.03	7	-	-0.17	-1.3	-3.4
(Acasta)	TTG	AG09-09	3600	-0.47	0.03	5	-	-0.01	-	-2.9
	TTG	AG09-16	3975	-0.25	0.04	5	-	0.01	-2.2	-2.0
Superior Province	TTG	INO5003	3784	-0.85	0.03	9	9.35	-0.04	1.6	0.8
(Nuvvuagittuq)	TTG	INO5012	3818	-0.68	0.04	7	10.6	-0.03	2.5	1.2
Kaapvaal Craton	TTG	KV2.1	3227	0.03	0.04	5	-	-0.06	-1.5	2.1
	TTG	NLS5.1	3236	-0.28	0.08	7	-	0.08	-2.0	2.2
	TTG	STEY1.5	3510	-0.24	0.04	5	7.41	0.05	3.0	2.4
	TTG	ST-J6	3429	-0.36	0.07	5	6.36	0.02	-0.5	-0.3
Austral Volcanic Zone	Adakite	Burney-2	0	-0.22	0.04	5	6.33	-0.05	-	-
	Adakite	Burney-4	0	-0.17	0.10	7	6.31	-0.08	-	-
	Adakite	Reclus-1	0	-0.25	0.03	5	7.45	-0.06	-	-
	Adakite	Viedma	0	-0.24	0.07	7	7.97	-0.06	-	-
Standards	And.	AGV-2	-	-0.23	0.02	9	-	-	-	-
	Dol.	W2-a	-	-0.16	0.03	18	-	-	-	-
	Carb.	SRM915b	-	-0.25	0.02	10	-	-	-	-

Bulk-silicate Earth (BSE) δ^{44}Ca = +0.95‰ (rel. to SRM915a) or +0.25‰ (rel. to SRM915b)[27]; Averages combine measurements using either SRM915a and/or SRM915b as bracketing standards; 2SE = 2SD/($n^{0.5}$). Si isotopic data are from Ref. 2. Oxygen isotopic data are from Refs. [2,3,160]. Neodymium and hafnium isotopic data are from [6]. U-Pb age estimates are from Refs. [2,116,161–165]. *And. = andesite, Dol. = dolerite, Carb. = carbonate.* For individual measurement results and alternate data normalizations, see Supplementary Data 3.

Supplementary Table 2.

Reduced partition function ratios (RPFR, $1000\ln\beta_{44/40}$) used in Ca isotope phase equilibrium modeling.

	Subtype	$1000\ln\beta_{44/40}$ (1000 K)	Notes	Reference
Clinopyroxene	Diopside	1.3113	DFT estimate	a
Hornblende	-	1.3113	Bond lengths & coordination ≈ Diopside	a-f
Epidote	-	1.0560	Bond lengths & coordination ≈ Anorthite	c, g, h
titanite	-	1.5083	Bond lengths & coordination ≈ Lime	c, i, j
Orthopyroxene	Enstatite	1.8465	DFT estimate, Ca/(Ca+Mg) = 1/64	a
Olivine	Forsterite	2.1094	DFT estimate, Ca/(Ca+Mg) = 1/64	a
Garnet	Almandine	1.8481	DFT estimate, Ca/(Ca+Fe) = 1/12	a
Plagioclase	Labradorite (An_{50})	0.9767	DFT estimate	a
K-Feldspar	-	0.9767	Assumed ≈ An_{50}	-
Silicate melt	-	1.1440	Empirical estimate ≈ (An_{50} + Diopside)/2	b-d, k
Mica	Muscovite & biotite	1.1440	Assumed ≈ silicate melt	-

[a] Antonelli et al., 2019 (EPSL)[19]; [b] Wang et al., 2019 (GCA)[29]; [c] Antonelli & Simon, 2020 (Chem. Geo.)[27]; [d] Antonelli et al., 2019 (PNAS)[26]; [e] Hawthorne & Oberti, 2007 (RiMG)[31]; [f] Zhou et al., 2016 (AGU abs.)[32]; [g] Brown et al., 2020 (EPSL)[34]; [h] Franz & Liebscher, 2004 (RiMG)[33]; [i] Kunz et al., 2000 (Am. Min.)[37]; [j] Hollabaugh & Foit, 1984 (Am. Min)[36]; [k] Zhang et al., 2018 (G-Cubed)[38].
