# Supplementary material for: Calcium isotope evidence for early Archaean carbonates and subduction of oceanic crust
Source: Nat Commun. 2021 May 5;12:2534. doi: 10.1038/s41467-021-22748-2 (PMC8099908; doi:10.1038/s41467-021-22748-2)
Supplement: Supplementary file 3 — Description of Additional Supplementary Files [file 41467_2021_22748_MOESM3_ESM.pdf]

Description of Additional Supplementary Files

Supplementary Data 1.
Major and trace-element results.
Supplementary Data 2.
Petrographic descriptions.
Supplementary Data 3.
Ca isotope data and alternate normalizations.
Supplementary Data 4.
Phase-equilibrium model results.
Supplementary Data 5.
Average carbonate A/CNK values (EarthChem).
